# Supplementary material for: S100A8/A9 as a prognostic biomarker with causal effects for post-acute myocardial infarction heart failure
Source: Nat Commun. 2024 Mar 27;15:2701. doi: 10.1038/s41467-024-46973-7 (PMC10973499; doi:10.1038/s41467-024-46973-7)

Supplementary Information

S100A8/A9 as a Prognostic Biomarker with Causal Effects for Post-acute Myocardial Infarction Heart Failure

Jie Ma, Yang Li, Ping Li, et, al.

CONTENTS

Supplementary Table 1 ... 03
Supplementary Table 2 ... 04
Supplementary Table 3 ... 05
Supplementary Table 4 ... 06
Supplementary Table 5 ... 08
Supplementary Table 6 ... 09
Supplementary Table 7 ... 13
Supplementary Table 8 ... 15
Supplementary Table 9 ... 16
Supplementary Table 10 ... 17
Supplementary Table 11 ... 18
Supplementary Table 12 ... 19
Supplementary Table 13 ... 20
Supplementary Table 14 ... 23
Supplementary Table 15 ... 26
Supplementary Table 16 ... 27
Supplementary Table 17 ... 28
Supplementary Fig. 1 ... 29
Supplementary Fig. 2 ... 30
Supplementary Fig. 3 ... 31
Supplementary Fig. 4 ... 32
Supplementary Fig. 5 ... 33
Supplementary Fig. 6 ... 34
Supplementary Fig. 7 ... 35
Supplementary Fig. 8 ... 36
Supplementary Fig. 9 ... 37
Supplementary Fig. 10 ... 38
Supplementary Fig. 11 ... 39
Supplementary Fig. 12 ... 40
Supplementary Fig. 13 ... 41
Supplementary Fig. 14 ... 42
Supplementary Fig. 15 ... 43
Supplementary Fig. 16 ... 44

Supplementary Table 1. Clinicopathological characteristics of patients with acute myocardial infarction (AMI) (n = 20) and healthy controls (HCs) (n = 10) in the first screening stage

Variable	HC (n=10)	AMI (n =20)		*P*-value
		No-HF events (n=10)	HF events (n=10)	
Age (years)	63.0 (54.8–71.5)	61.0 (54.8–70.8)	63.0 (54.8–71.5)	0.950
Male sex	6 (60.0)	6 (60.0)	6 (60.0)	1.000
SBP (mm Hg)	118.5 (112.8–124.3)	116.5 (108.0–125.3)	111.5 (101.0–120.5)	0.168
DBP (mm Hg)	78.0 (72.8–83.0)	77.0 (73.5–81.5)	71.5 (67.3–82.3)	0.388
Current smoking	5 (50.0)	5 (50.0)	6(60.0)	1.000
Max Killip classification				/
Ⅰ	/	10 (100.0)	0 (0.0)	
Ⅱ	/	0 (0.0)	0 (0.0)	
Ⅲ	/	0 (0.0)	7 (70.0)	
Ⅳ	/	0 (0.0)	3 (30.0)	
Hypertension	3 (30.0)	6 (60.0)	5 (50.0)	0.260
Hyperlipidaemia	3 (30.0)	6 (60.0)	8 (80.0)	0.056
Diabetes mellitus	1 (10.0)	2 (20.0)	44 (40.0)	0.372
Culprit lesion profiles				/
LAD	/	4 (40.0)	4 (40.0)	1.000
LCX	/	3 (30.0)	2 (20.0)	1.000
RCA	/	3 (30.0)	4 (40.0)	1.000
PCI	/	10 (100.0)	10 (100.0)	/

AMI, acute myocardial infarction; DBP, diastolic blood pressure; HC, healthy controls; HF, heart failure; LAD, left anterior descending artery; LCX, left circumflex artery; RCA, right coronary artery; SBP, systolic blood pressure; PCI, percutaneous coronary intervention.

Data were presented as absolute numbers (percentages) or medians (interquartile ranges). Categorical variables were analyzed using a two-sided χ^2 or Fisher's exact test. Continuous parametric data were analyzed using a two-sided t-test. Continuous non-parametric data were analyzed using a two-sided the Mann–Whitney U test. $P < 0.05$ was considered as significant. Source data are provided in the Source Data File.

Supplementary Table 2. Occurrence of adverse events in the discovery cohort and validation cohort during the follow-up period

cohort	HF events					Cardiac death	Non-cardiac death
	In-hospital			Long-term			
	new HF onset	CS	Death due to HF/CS	HF progression resulting in re-hospitalization	Death due to HF	Insufficient evidence to determine death from HF	Neoplasms, traffic accident, etc
Discovery (n=1062)	66	35	17	112	66	18	3
Validation (n=1043)	70	30	10	66	16	2	1

CS, cardiogenic shock ; HF, heart failure

Supplementary Table 3. Definitions of the heart failure (HF) events

Outcome		Definition
in-hospital	new HF onset	The new onset of signs and symptoms of HF were clinical manifestations such as dyspnea, orthopnea, peripheral edema, jugular vein dilatation, a third heart sound (S3), and lung rale.
	worsening HF	Killip class II progressed to III or IV, Killip class III progressed to IV
	cardiogenic shock	systolic blood pressure ≤90 mm Hg for > 30 min after exclusion of hypovolaemia, with clinical evidence of hypoperfusion, inotrope dependence, or mechanical left ventricular support to correct the issue.
	Death due to HF or cardiogenic shock	Death due to HF or cardiogenic shock was defined as a death with clinical, radiologic, or postmortem evidence of HF, in the absence of in-hospital acute ischemic events.
Long-term	HF progression resulting in re-hospitalization	A hospital readmission for which HF was the primary reason, specifically defined as an event meeting all of the following criteria: (1) admission to the hospital for at least 24 hours; (2) objective evidence of new or worsening HF (e.g., orthopnea, jugular venous distension, pulmonary basilar crackles, etc.); and (3) intensification of HF therapy (e.g., initiation of intravenous diuretics or inotropes).
	Death due to HF	Death due to HF was defined as death in the context of clinically worsening symptoms and/or signs of HF with no other apparent cause, death as a consequence of a surgical procedure to treat HF, or death after referral to hospice for HF.

HF, heart failure

Supplementary Table 4. Comparison of baseline information between the discovery cohort and the validation cohort

Variables	Discovery cohort (n=1062)	Validation cohort (n=1043)	*P*-value
**Demographics**			
Age (years)	59.0 (51.0-67.0)	40.0 (36.0-42.0)	**1.74E-269**
Male sex	851 (80.1)	951 (91.2)	**5.23E-13**
SBP (mm Hg)	120.0 (109.0-135.0)	125.0 (117.0-133.0)	**1.12E-05**
DBP (mm Hg)	73.0 (68.0-82.0)	79.0 (70.0-84.0)	**7.57E-11**
Current smoking	652 (61.4)	680 (65.2)	**0.070**
STEMI	796 (75.0%)	642 (61.6%)	**3.93E-11**
Killip classification			
I	879 (82.8)	971 (93.1)	**3.84E-13**
II	163 (15.3)	58 (5.6)	**2.40E-13**
III	20 (1.9)	14 (1.3)	0.325
**Medical history**			
Hypertension	621 (58.5)	513 (49.2)	**1.90E-05**
Hyperlipidemia	695 (65.4)	482 (46.2)	**6.41E-19**
Diabetes mellitus	355 (33.4)	213 (20.4)	**1.80E-11**
CAD	258 (24.3)	209 (20.0)	**0.019**
**Biochemical**			
Neutrophil counts ($\times10^9$/L)	5.8 (4.6-7.9)	6.3 (4.7-8.3)	**0.002**
HDL cholesterol (mmol/L)	1.0 (0.9-1.2) [†]	0.9 (0.8-1.1)	**3.98E-19**
LDL cholesterol (mmol/L)	2.8 (2.3-3.5) [†]	2.3 (1.7-3.0)	**6.96E-32**
Fasting glucose (mmol/L)	6.7 (5.6-9.3)	5.5 (5.0-6.6)	**5.30E-51**
Creatinine (μmol/L)	73.3 (62.8-84.5)	71.3 (63.8-79.5)	**0.004**
**Biomarkers**			
cTnI (ng/mL)	1.5 (0.2-9.6)	1.2 (0.2-6.6)	0.095
BNP (pg/mL)	107.7 (42.0-243.2)	82.0 (28.0-189.4)	**8.80E-08**
hs-CRP (mg/L)	6.0 (2.1-19.1)	4.7 (1.7-13.5)	**8.00E-05**
S100A8/A9 (ng/ml)	3640.8 (2345.7-5068.8)	3657.4 (1905.0-4877.0)	0.060
**Overall lesion profiles**			
Left main artery disease	32 (3.0)	40 (3.8)	0.300
2-vessel disease	311 (29.3)	192 (18.4)	**4.91E-09**
3-vessel disease	137 (12.9)	226 (21.7)	**1.01E-07**
**Echocardiography**			
Admission LVEF (%)	54.0 (48.0-58.0)	55.0 (50.0-60.0)	**6.80E-06**
**Infarct size**			
CK-MB-based estimation (ng*h/ml)	5022.3 (2014.6-7088.9)	4456.1 (2319.1-6275.4)	**0.002**
**Medication at discharge**			
Aspirin	1038 (97.7)	1010 (96.8)	**3.20E-05**
P2Y12 receptor Inhibitor	1062 (100.0)	964 (92.4)	**1.19E-19**
Statin	1019 (96.0)	990 (94.9)	**0.002**
ACEI or ARB	606 (57.1)	449 (43.0)	**8.62E-12**
Beta-blockers	783 (73.7)	729 (69.9)	**0.010**
MRA	175 (16.5)	70 (6.7)	**1.76E-11**

ACEI, angiotensin-converting enzyme inhibitor; ARB, angiotensin-receptor blocker; BNP, B-type natriuretic peptide; CAD, coronary artery disease; cTnI, cardiac troponin I; DBP, diastolic blood pressure; HDL, high-density lipoprotein; hs-CRP, high-sensitivity C-reactive protein; LDL, low-density lipoprotein; LVEF, left ventricular ejection fraction; MRA, mineralocorticoid receptor antagonist; P2Y12 receptor

Inhibitor included clopidogrel and ticagrelor; SBP, systolic blood pressure; STEMI, ST-segment myocardial infraction.

Data were presented as absolute numbers (percentages) or medians (interquartile ranges). Categorical variables were analyzed using a two-sided χ^2 or Fisher's exact test. Continuous parametric data were analyzed using a two-sided t-test. Continuous non-parametric data were analyzed using a two-sided the Mann–Whitney U test. P < 0.05 was considered as significant. Source data are provided in the Source Data File. Bold indicated P<0.05. †1–4% data missing.

Supplementary Table 5. Characteristics of the HCs (n = 588)

Variable	HCs (n = 588)	
	**Female (n = 139)**	**Male (n = 449)**
Age (years)	49.0 (44.0–55.0)	48.0 (43.0–53.5)
BMI	23.7 (22.2–25.7)	25.3 (23.1–27.6)
SBP (mm Hg)	122.0 (112.0–133.0)	124.0 (115.0–133.0)
DBP (mm Hg)	79.0 (70.0–87.0)	83.0 (75.5–89.0)
Current smoking	0 (0.0)	225 (50.1)
HDL cholesterol (mmol/L)	1.4 (1.2–1.7)	1.2 (1.1–1.4)
LDL cholesterol (mmol/L)	3.1 (2.7–3.7)	3.2 (2.8–3.7)
Fasting glucose (mmol/L)	5.3 (5.0–5.6)	5.3 (5.0–5.6)

BMI, Body Mass Index; DBP, diastolic blood pressure; HC, healthy controls; LDL, low-density lipoprotein; SBP, systolic blood pressure. Current smokers refer to those who have smoked 100 cigarettes in their lifetime and had smoked cigarettes in the past 30 days.

Supplementary Table 6. Common differential proteins across the three groups

Protein abbreviation	Full name of each protein	AMI vs. HC		HF events vs. No-HF events	
		log_2FC	P-value	log_2FC	P-value
S100A12	S100 calcium-binding protein A12	6.201	4.66E-07	3.698	1.08E-05
S100A8	S100 calcium-binding protein A8	4.702	1.33E-07	2.666	1.08E-05
S100 A8/A9	S100 calcium-binding proteins A8/A9	3.249	6.66E-08	2.030	1.08E-05
SNCG	Synuclein gamma	-2.184	3.29E-05	-1.412	2.17E-05
S100A6	S100 calcium-binding protein A6	2.688	7.99E-07	1.546	1.30E-04
CCK	Cholecystokinin	1.017	4.46E-06	0.667	1.30E-04
SART3	Spliceosome-associated factor 3	1.284	5.79E-05	0.912	2.06E-04
BNP	Natriuretic peptides B	1.518	4.38E-05	1.342	3.25E-04
Cadherin-13	Cadherin-13	2.047	9.83E-05	1.647	4.87E-04
pro-MMP13	Pro-Matrix metalloproteinase-13	1.992	5.79E-05	1.179	7.25E-04
Visfatin	Nicotinamide phosphoribosyltransferase	1.055	4.38E-05	0.708	7.25E-04
SEMA3A	Semaphorin-3A	1.287	6.16E-04	1.046	1.05E-03
C5/C5a	Complement C5	1.629	2.59E-04	0.915	1.05E-03
ApoA1	Apolipoprotein A-I	0.826	4.04E-04	0.582	1.05E-03
ALBUMIN	Albumin	0.587	6.16E-04	0.464	1.05E-03
Syndecan-3	Syndecan-3	1.187	3.84E-03	1.191	1.50E-03
TRAIL/ TNFSF10	Tumor necrosis factor ligand superfamily member 10	1.137	2.75E-03	0.926	1.50E-03
Caspase-3	Caspase-3	1.361	2.44E-05	0.852	1.50E-03
Plasminogen	Plasminogen	1.026	4.04E-04	0.763	1.50E-03
TCCR / WSX-1	T-cell cytokine receptor	1.083	2.00E-06	0.683	1.50E-03
PAK7	P21-activated kinase 7	1.131	1.90E-02	1.249	2.09E-03
FoxO1	Forkhead box protein O1	0.910	4.38E-05	0.659	2.09E-03
Neuritin	Neuritin	1.596	1.62E-04	0.292	2.09E-03
DCBLD2	CUB and LCCL domain containing 2	0.795	1.67E-02	0.829	2.88E-03
Insulysin / IDE	Insulysin	1.238	9.25E-06	0.777	2.88E-03
PYK2	Proline-rich tyrosine kinase 2	1.044	9.25E-06	0.769	2.88E-03
Kremen-2	Kremen protein 2	1.000	5.79E-05	0.624	2.88E-03
LBP	Lipopolysaccharide-binding protein	0.916	6.46E-06	0.567	2.88E-03
Vitamin D-BP	Vitamin D-binding protein	0.925	5.00E-04	0.663	3.89E-03
TLR2	Toll-like receptor 2	1.412	4.46E-06	0.438	3.89E-03
HSP90	Heat shock protein 90	-1.589	2.59E-04	-0.761	3.89E-03
Troponin I	Troponin I1	2.411	4.46E-06	2.346	5.20E-03
Lipocalin-2	Lipocalin-2	1.512	4.46E-06	0.857	5.20E-03
IL-17RD	Interleukin-17 receptor D	0.720	2.05E-04	0.494	5.20E-03
Vitronectin	Vitronectin	-1.356	1.62E-03	-1.161	5.20E-03
Serpin A9	Serpin A9	-0.901	1.67E-02	-1.735	5.20E-03
IL-4 R	Interleukin-4 receptor	1.006	2.05E-04	0.672	6.84E-03
Angiostatin	Angiostatin	0.564	9.83E-05	0.349	6.84E-03
6Ckine	C-C motif chemokine 21	-0.448	2.75E-03	-2.645	6.84E-03
CXCR2 / IL-8 RB	C-X-C chemokine receptor type 2	1.099	3.29E-05	0.702	8.93E-03
IL-23 R	Interleukin-23 receptor	1.092	7.57E-05	0.677	8.93E-03
IBSP	Integrin-binding sialoprotein	0.935	1.26E-04	0.634	8.93E-03

Aldolase A	Aldolase A	0.790	1.62E-04	0.531	8.93E-03
Progranulin	Progranulin	0.724	5.79E-05	0.432	8.93E-03
Kallikrein 6	Kallikrein-6	0.554	1.35E-03	0.406	8.93E-03
ADAMTS-18	A disintegrin and metalloproteinase with thrombospondin type-1 modules 18	-1.345	1.62E-04	-0.855	8.93E-03
BAF57	BRG1-associated factor 57	-0.874	2.76E-02	-1.473	8.93E-03
Pappalysin-1	Pappalysin-1	-1.698	2.32E-03	-2.115	8.93E-03
CK-MB	Creatine kinase B-type	2.190	3.29E-05	0.946	1.15E-02
FABP1	Fatty acid-binding protein	1.219	2.00E-06	0.831	1.15E-02
TFF3	Trefoil factor 3	1.175	1.62E-04	0.751	1.15E-02
TL1A / TNFSF15	Tumor necrosis factor ligand superfamily member 15	1.143	3.29E-05	0.735	1.15E-02
FADD	FAS-associated death domain protein	0.986	4.38E-05	0.634	1.15E-02
Endocan	Endocan	0.951	5.00E-04	0.613	1.15E-02
EGF R / ErbB1	Epidermal growth factor receptor	0.874	9.83E-05	0.497	1.15E-02
CART	Cocaine- and amphetamine-regulated transcript protein	-0.754	7.18E-03	-0.335	1.15E-02
DLL1	Delta-like protein 1	-1.158	2.75E-03	-0.608	1.15E-02
LIF	Leukemia inhibitory factor	1.053	2.76E-02	1.066	1.47E-02
EMAP-II	Endothelial monocyte activating polypeptide II	1.454	4.38E-05	0.862	1.47E-02
EDAR	Ectodysplasin A receptor	1.335	1.26E-04	0.830	1.47E-02
PGRP-S	Peptidoglycan recognition protein-S	1.127	2.59E-04	0.820	1.47E-02
HSP20	Heat shock protein 20	1.590	4.38E-05	0.809	1.47E-02
sFRP-1	Secreted frizzled-related protein 1	1.048	5.79E-05	0.686	1.47E-02
ICAM-3 (CD50)	Intercellular adhesion molecule 3	1.023	1.26E-04	0.638	1.47E-02
TMEFF2	Transmembrane protein with EGF-like and two follistatin-like domains 2	0.839	1.94E-03	0.618	1.47E-02
IL-20 R beta	Interleukin-20 receptor subunit beta	0.820	5.79E-05	0.564	1.47E-02
GASP-1 / WFIKKNRP	Kunitz and NTR domain-containing-related protein	0.708	1.26E-04	0.403	1.47E-02
ADAMTS-5	A disintegrin and metalloproteinase with thrombospondin motifs 5	-3.190	5.00E-04	-1.694	1.47E-02
B3GNT1	UDP-GlcNAc:betaGal beta-1,3-N-acetylglucosaminyltransferase 2	-1.335	1.62E-03	-2.150	1.47E-02
FX	Coagulation factor X	1.613	7.54E-04	1.132	1.85E-02
TLR3	Toll-like receptor 3	1.311	1.12E-03	0.553	1.85E-02
CRTH-2	Chemoattractant receptor-homologous molecule expressed on Th2 cells	1.076	1.79E-05	0.537	1.85E-02
NrCAM	Neuronal cell adhesion molecule	0.865	7.54E-04	0.522	1.85E-02
EGF	Epidermal growth factor	0.802	5.00E-04	0.504	1.85E-02
NRG1 Isoform GGF2	Neuregulin1 isoform GGF2	1.473	3.29E-05	0.504	1.85E-02
Aldolase C	Aldolase C	0.665	1.12E-03	0.484	1.85E-02
ICAM-5	Intercellular adhesion molecule 5	0.666	2.59E-04	0.418	1.85E-02
VEGI/ TNFSF15	Tumor necrosis factor ligand superfamily member 15	1.719	8.32E-03	1.218	2.32E-02
PDGF-C	Platelet-derived growth factor C	1.164	1.12E-03	0.687	2.32E-02
KLF4	Krueppel-like factor 4	0.833	1.26E-04	0.545	2.32E-02
TLR1	Toll-like receptor 1	0.884	1.62E-04	0.534	2.32E-02
Nesfatin-1	Nesfatin-1	0.691	2.32E-03	0.483	2.32E-02
Beta Defensin 4	Beta-defensin 4A	0.672	9.20E-04	0.476	2.32E-02
pro-Glucagon	Pro-Glucagon	0.751	4.04E-04	0.466	2.32E-02

Nidgen-1	Nidogen-1	0.710	7.57E-05	0.446	2.32E-02
ApoB100	Apolipoprotein B-100	1.028	4.51E-03	0.443	2.32E-02
CXCR1 / IL-8 RA	C-X-C chemokine receptor type 1	0.787	3.24E-04	0.432	2.32E-02
IL-34	Interleukin-34	0.947	9.20E-04	0.359	2.32E-02
IGF-II	Insulin-like growth factor II	0.541	3.84E-03	0.334	2.32E-02
EphB1	Ephrin type-B receptor 1	0.462	1.90E-02	0.235	2.32E-02
RIP1	Receptor Interacting Protein 1	-1.387	5.00E-04	-1.189	2.32E-02
MTUS1	Microtubule-associated tumor suppressor 1	1.373	1.67E-02	1.551	2.88E-02
AMICA	Adhesion molecule interacting with CXADR antigen 1	1.114	2.75E-03	1.327	2.88E-02
CRP	C-reactive protein	1.396	7.57E-05	0.889	2.88E-02
HVEM / TNFRSF14	Tumor necrosis factor receptor superfamily member 14	0.056	1.27E-02	0.807	2.88E-02
LOX-1	Lectin-like oxidized low-density lipoprotein receptor-1	1.502	7.54E-04	0.730	2.88E-02
FGFR1	Fibroblast growth factor receptor 1	0.928	1.62E-04	0.571	2.88E-02
Galectin-3BP	Galectin-3-binding protein	0.872	4.04E-04	0.567	2.88E-02
LTK	Leukocyte tyrosine kinase	0.426	3.84E-03	0.319	2.88E-02
NCAM-1 / CD56	Neural cell adhesion molecule 1	0.555	1.35E-03	0.318	2.88E-02
EphA7	Ephrin type-A receptor 7	0.509	9.62E-03	0.244	2.88E-02
Fc RIIB/C	Fc fragment of IgG receptor IIb	0.304	4.90E-02	-0.052	2.88E-02
S-100b	Protein S100-B	-0.864	3.11E-02	-0.491	2.88E-02
Chemerin	Chemerin	2.182	3.11E-02	-0.781	2.88E-02
AMPKa1	Protein kinase AMP-activated catalytic subunit alpha 1	-0.964	2.32E-03	-0.945	2.88E-02
TRPC6	Transient receptor potential channel 6	-1.754	2.05E-04	-3.745	2.88E-02
cTnT	Cardiac troponin T	1.920	6.66E-08	1.137	3.55E-02
PSP	Prostate secretory protein	1.150	9.20E-04	0.864	3.55E-02
ADAMTS-13	a disintegrin and metalloproteinase with a thrombospondin type 1 motif, member 13	1.200	5.00E-04	0.831	3.55E-02
Glypican 3	Glypican-3	0.656	7.18E-03	0.523	3.55E-02
MMP-16 / MT3-MMP	Matrix metalloproteinase-16	0.987	1.12E-03	0.476	3.55E-02
CXCR6	C-X-C chemokine receptor type 6	0.647	1.12E-03	0.416	3.55E-02
IRF6	Interferon regulatory factor 6	0.708	1.12E-03	0.403	3.55E-02
Angiopoietin-like 1	Angiopoietin-like 1	0.514	2.05E-04	0.296	3.55E-02
Attractin	Attractin	0.452	5.00E-04	0.296	3.55E-02
Midkine	Midkine	1.184	1.27E-02	0.142	3.55E-02
RET	Ret proto-oncogene	-1.357	3.26E-03	-0.869	3.55E-02
Insulin R	Insulin receptor	-1.190	9.20E-04	-1.098	3.55E-02
Activin A	Activin A	-0.779	4.04E-04	-4.920	3.55E-02
Cathepsin B	Cathepsin B	1.979	6.16E-04	0.878	4.33E-02
FOLR1	Folate receptor alpha	1.994	7.57E-05	0.851	4.33E-02
VEGF R2(KDR)	kinase insert domain receptor	0.747	3.11E-02	0.738	4.33E-02
Angiogenin	Angiogenin	1.463	1.26E-04	0.672	4.33E-02
MFRP	Membrane frizzled-related protein	0.751	4.04E-04	0.470	4.33E-02
EphB6	Ephrin type-B receptor 6	0.824	6.16E-04	0.447	4.33E-02
Thrombospondin-4	Thrombospondin-4	0.969	6.16E-04	0.442	4.33E-02
IL-27	Interleukin-27 subunit alpha	0.703	1.26E-04	0.438	4.33E-02

TRKB	Tropomyosin-related kinase B	0.725	4.51E-03	0.433	4.33E-02
Kallikrein 14	Kallikrein-14	0.704	2.76E-02	0.241	4.33E-02
Survivin	Survivin	1.155	4.39E-02	-0.073	4.33E-02
Tarc	Thymus and activation-regulated chemokine	-1.152	3.84E-03	-0.949	4.33E-02
TAF4	TATA-box binding protein associated factor 4	-1.397	1.35E-03	-1.037	4.33E-02
Desmin	Desmin	-2.533	2.59E-04	-1.843	4.33E-02
B7-1 /CD80	CD80 molecule	-1.026	1.26E-04	-3.013	4.33E-02

AMI, acute myocardial infarction; HC, healthy controls; HF, heart failure. Differences between the two groups were analyzed using a two-sided Wilcoxon-test. $P < 0.05$ was considered as significant. P-values were not corrected for multiple testing. Source data are provided in the Source Data File.

Supplementary Table 7. Univariable predictors of post-AMI HF events in the discovery cohort

Variable	Discovery cohort (n = 1,062)	
	Crude HR (95%CI)	*P*-value
**Demographic**		
Age*	1.27 (1.12–1.43)	**1.43E-04**
Male sex	0.73 (0.56–0.96)	**0.022**
SBP*	0.77 (0.67–0.88)	**8.60E-05**
DBP*	0.78 (0.69–0.89)	**2.08E-04**
Current smoking	1.10 (0.87–1.39)	0.429
Killip classification >1	1.48 (1.13–1.95)	**0.005**
**Medical history**		
Hypertension	1.02 (0.81–1.29)	0.849
Hyperlipidaemia	1.14 (0.89–1.45)	0.304
Diabetes mellitus	1.10 (0.87–1.40)	0.415
CAD	1.05 (0.82–1.35)	0.680
**Routine biochemical at admission**		
Neutrophil count*	1.38 (1.22–1.56)	**4.58E-07**
HDL cholesterol*	1.02 (0.89–1.16)	0.831
LDL cholesterol*	0.99 (0.87–1.13)	0.911
Fasting glucose*	1.23 (1.07–1.41)	**0.004**
Creatinine*	1.32 (1.11–1.57)	**0.002**
**Biomarkers at admission**		
cTnI*	1.39 (1.19–1.63)	**2.70E-05**
BNP*	1.61 (1.38–1.87)	**7.19E-10**
hs-CRP*	1.25 (1.10–1.43)	**0.001**
S100A8/A9*	1.92 (1.69–2.19)	**3.95E-23**
S100A12*	1.09 (0.97–1.24)	0.157
**Echocardiography**		
Admission LVEF*	0.62 (0.55–0.70)	**1.76E-13**
**Infarct size**		
CK-MB-based estimation (ng*h/ml) *	1.24 (1.10,1.40)	**4.42E-04**
**Overall lesion profiles**		
Multi-vessel disease	1.18 (0.94–1.48)	0.161
Left main artery disease	2.18 (1.30–3.67)	**0.003**
**At discharge**		
Aspirin	0.52 (0.17–1.63)	0.265
Statin	0.96 (0.45–2.03)	0.914
ACEI or ARB	0.97 (0.77–1.23)	0.820
Beta-blockers	1.20 (0.91–1.60)	0.203
MRA	0.88 (0.64-1.23)	0.455

*Expressed as per one standard deviation increase.

ACEI, angiotensin-converting enzyme inhibitor; ARB, angiotensin-receptor blocker; BNP, B-type natriuretic peptide; CAD, coronary artery disease; CI, confidence interval; cTnI, cardiac troponin I; DBP, diastolic blood pressure; HDL, high-density lipoprotein; HR, hazard ratio; hs-CRP, high-sensitivity C-reactive protein; LDL, low-density lipoprotein; LVEF, left ventricular ejection fraction; MRA, mineralocorticoid receptor antagonist; P2Y12 receptor Inhibitor included clopidogrel and ticagrelor; SBP, systolic blood pressure.

Cox regression was used to evaluate the association of variables and HF events. All tests were two-sided and $P < 0.05$ was considered as significant. Bold indicated $P<0.05$.

Supplementary Table 8. C-statistics of clinical biomarkers and candidate proteins for HF events in the discovery cohort

Biomarkers	C-statistic (95% CI)	*P*-value
S100A8/A9	0.65 (0.62–0.68)	8.39E-20
S100A12	0.53 (0.49–0.56)	0.108
cTnI	0.56 (0.53–0.60)	2.41E-04
CRP	057 (0.53–0.60)	1.67E-04
BNP	0.61 (0.57–0.64)	3.61E-10

BNP, B-type natriuretic peptide; CRP, C-reactive protein; cTnI, cardiac troponin I. Harrell's concordance C-statistic was used to compare the predictive accuracy of the candidate proteins and clinical biomarkers. All tests were two-sided and $P < 0.05$ was considered as significant. Source data are provided in the Source Data File.

Supplementary Table 9. Three-categories net reclassification index (NRI) of S100A8/A9 and S100A12 for HF events on the existing models in the discovery cohort

Reference model	S100A8/A9			S100A12		
	< 0.1	0.1–0.3	≥ 0.3	< 0.1	0.1–0.3	≥ 0.3
Total population						
< 0.1	60	25	0	75	10	0
0.1-0.3	139	330	125	5	576	13
≥ 0.3	13	83	287	0	19	364
HF events						
< 0.1	8	9	0	16	1	0
0.1-0.3	8	53	44	0	104	1
≥ 0.3	0	19	155	0	4	170
Number of correct reclassifications		26			-2	
Non-HF events						
< 0.1	52	16	0	59	9	0
0.1–0.3	131	277	81	5	472	12
≥ 0.3	13	64	132	0	15	194
Number of correct reclassifications		111			-1	

The patients were divided into subgroups that did or did not reach the primary endpoint of HF events.

The number of patients in each risk category was shown.

HF, heart failure; NRI, net reclassification improvement.

Supplementary Table 10. C-statistics of clinical biomarkers and candidate proteins for HF events in the validation cohort

Biomarkers	C-statistic (95% CI)	*P*-value
S100A8/A9	0.70(0.66–0.74)	4.75E-22
cTnI	0.60 (0.55–0.64)	1.65E-05
CRP	0.57 (0.53–0.61)	0.001
BNP	0.67 (0.63–0.71)	7.75E-18

BNP, B-type natriuretic peptide; CRP, C-reactive protein; cTnI, cardiac troponin I. Harrell's concordance C-statistic was used to compare the predictive accuracy of the candidate proteins and clinical biomarkers. All tests were two-sided and $P < 0.05$ was considered as significant.

Supplementary Table 11. Three-categories NRI of S100A8/A9 for HF events on the existing models in the validation cohort

**Reference model**	**S100A8/A9**		
	< 0.1	0.1–0.3	≥ 0.3
Total population			
< 0.1	410	68	0
0.1–0.3	115	224	42
≥ 0.3	3	26	155
HF events			
< 0.1	17	12	0
0.1–0.3	12	31	17
≥ 0.3	0	7	96
Number of correct reclassifications		10	
Non-HF events			
< 0.1	393	56	0
0.1–0.3	103	193	25
≥ 0.3	3	19	59
Number of correct reclassifications		44	

The patients were divided into subgroups that did or did not reach the primary endpoint of HF events. The number of patients in each risk category was shown.

HF, heart failure; NRI, net reclassification improvement.

Supplementary Table 12. Association of S100A8/A9 and HF risk before and after January 1st 2020

	Before COVID-19		After COVID-19	
	HR (95%CI)	*P-value*	HR (95%CI)	*P-value*
Discovery cohort (n=1062)				
S100A8/A9	1.71 (1.41-2.07)	5.43E-08	3.65 (2.54-5.24)	2.67E-12
Adjusted for model 1	1.79 (1.47-2.17)	7.03E-09	3.86 (2.66-5.60)	1.16E-12
Adjusted for model 2	1.83 (1.50-2.24)	3.76E-09	3.96 (2.70-5.81)	1.96E-12
Adjusted for model 3	1.76 (1.43-2.17)	7.37E-08	4.22 (2.81-6.31)	3.01E-12
Validation cohort (n=1043)				
S100A8/A9	2.91 (2.07-4.09)	8.78E-10	1.99 (1.28-3.11)	0.002
Adjusted for model 1	2.91 (2.06-4.10)	1.05E-09	1.96 (1.25-3.08)	0.003
Adjusted for model 2	2.80 (1.97-3.96)	6.85E-09	1.93 (1.23-3.03)	0.004
Adjusted for model 3	2.40 (1.68-3.42)	1.00E-06	1.95 (1.22-3.12)	0.005

Unadjusted and adjusted HRs from Cox proportional hazards regression analysis were shown for discovery-cohort (n=1062) and validation--cohort (n = 1043) patients with HF. S100A8/A9 was measured on admission. Model 1: adjusted for age and sex; model 2: adjusted for model 1+systolic blood pressure, Killip classification at admission, fasting glucose, creatinine, left main artery disease; model 3: adjusted for model 2+neutrophil count, cardiac troponin I, B-type natriuretic peptide, c-reactive protein, left ventricular ejection fraction at admission and estimated infarct size (CK-MB AUC_{0-72}). All tests were two-sided and $P < 0.05$ was considered as significant. HR, hazard ratio. Source data are provided in the Source Data File.

Supplementary Table 13. Baseline characteristics of the study population grouped by the quartiles of S100A8/A9

Variables	Discovery cohort (n=1062)					Validation cohort (n=1043)				
	**≤ 2346 (n = 265)**	**2346-3641 (n = 266)**	**3641-5069 (n= 266)**	**>5069 (n = 265)**	***P* for trend**	**≤ 1905 (n =261)**	**1905-3657 (n = 261)**	**3657-4877 (n= 261)**	**>4877 (n = 260)**	***P* for trend**
**Demographics**										
Age (years)	62.0 (54.0-69.0)	60.0 (50.0-68.0)	59.0 (52.0-67.3)	55.0 (48.0-64.0)	**5.00E-06**	39.0 (36.0-41.0)	39.0 (36.0-43.0)	40.0 (37.0-43.0)	40.0 (37.0-42.0)	0.196
Male sex	207 (78.1)	199 (74.8)	221 (83.1)	224 (84.5)	**0.012**	238 (91.2)	233 (89.3)	2843 (93.1)	237 (91.2)	0.365
SBP (mm Hg)	120.0 (107.0-132.0)	120.0 (110.0-135.0)	120.0 (110.0-137.0)	120.0 (107.0-135.0)	0.556	127.0 (117.5-136.0)	125.0 (119.0-132.0)	116.0 (122.0-131.0)	123.0 (116.0-130.8)	**0.010**
DBP (mm Hg)	70.0 (65.0-80.0)	72.0 (70.0-80.0)	73.0 (67.8-84.0)	75.0 (69.0-83.0)	**0.037**	79.0 (70.0-86.0)	80.0 (70.0-83.5)	78.0 (70.0-82.0)	79.0 (70.0-81.9)	0.430
Current smoking	149 (56.2)	163 (61.3)	162 (60.9)	178 (67.2)	0.015	184 (70.5)	161 (61.7)	173 (66.3)	162 (62.3)	0.130
Killip classification					0.332					**0.001**
I	225 (84.9)	218 (82.0)	219 (82.3)	217 (81.9)		249 (95.4)	252 (96.6)	235 (90.0)	235 (90.4)	
II	38 (14.3)	40 (15.0)	43 (16.2)	42 (15.8)		11 (4.2)	8 (3.1)	20 (7.7)	19 (7.3)	
III	2 (0.8)	8 (3.0)	4 (1.5)	6 (2.3)		1 (0.4)	1 (0.4)	6 (2.3)	6 (2.3)	
**Medical history**										
Hypertension	156 (58.9)	157 (59.0)	152 (57.1)	156 (58.9)	0.889	122 (46.7)	131 (50.2)	124 (47.5)	136 (52.3)	0.312
Hyperlipidemia	167 (63.0)	164 (61.7)	170 (63.9)	194 (73.2)	**0.012**	121 (46.4)	119 (45.6)	122 (46.7)	120 (46.2)	0.969
Diabetes mellitus	96 (36.2)	91 (34.2)	89 (33.5)	79 (29.8)	0.123	51 (19.5)	53 (20.3)	54 (20.7)	55 (21.2)	0.640
CAD	93 (30.2)	84 (24.4)	77 (24.1)	65 (18.5)	**0.006**	56 (21.5)	58 (22.2)	52 (19.9)	43 (16.5)	0.125
**Biochemical**										
Neutrophil counts ($\times 10^9$/L)	5.4 (4.1-7.1)	6.1 (4.4-8.3)	6.7 (5.1-8.6)	7.2 (5.3-9.3)	**2.89E-16**	5.2 (4.2-7.1)	5.7 (4.6-7.1)	5.9 (4.7-8.1)	6.8 (5.1-8.7)	**4.90E-12**
HDL cholesterol (mmol/L)	1.0 (0.9-1.2)	1.0 (0.9-1.2)	1.1 (0.9-1.2)	1.0 (0.9-1.2)	0.773	0.9 (0.8-1.1)	1.0 (0.8-1.1)	0.9 (0.8-1.1)	0.9 (0.8-1.1)	0.299
LDL cholesterol (mmol/L)	2.7 (2.3-3.4)	2.9 (2.3-3.4)	2.9 (2.2-3.5)	2.9 (2.2-3.4)	0.779	2.3 (1.8-3.2)	2.3 (1.8-3.0)	2.3 (1.7-2.9)	2.2 (1.8-3.0)	0.200
Fasting glucose (mmol/L)	6.7 (5.6-9.3)	6.4 (5.5-9.0)	6.7 (5.6-9.1)	7.2 (5.6-9.7)	0.543	5.6 (5.1-6.9)	5.4 (5.0-6.6)	5.5 (5.0-6.3)	5.4 (5.0-6.7)	0.604

Creatinine (μmol/L)	72.9 (63.2-85.4)	72.6 (61.9-84.2)	73.3 (62.4-83.3)	74.5 (62.8-85.3)	0.520	71.3 (63.0-78.6)	69.5 (64.6-78.8)	72.0 (63.8-80.0)	71.7 (64.8-81.5)	0.740
**Biomarkers**										
cTnI (ng/mL)	0.9 (0.1-5.4)	1.3 (0.1-9.5)	2.1 (0.2-14.3)	1.9 (0.2-11.5)	**0.024**	1.2 (0.2-6.5)	1.2 (0.2-4.6)	1.2 (0.3-6.6)	0.8 (0.1-9.1)	**0.001**
BNP (pg/mL)	101.0 (40.0-234.0)	100.0 (48.5-225.1)	110.0 (40.5-262.0)	127.0 (47.5-256.0)	0.581	78.6 (28.0-165.0)	83 (27.0-189.0)	75.0 (29.0-191.4)	96.6 (32.3-221.3)	**0.003**
hs-CRP (mg/L)	5.0 (1.8-15.8)	5.4 (1.9-19.9)	5.8 (2.2-17.5)	8.0 (2.7-21.9)	0.083	4.9 (1.6-15.4)	4.1 (1.5-11.7)	4.3 (1.7-12.9)	5.7 (1.9-15.5)	0.187
**Overall lesion profiles**										
Multi-vessel disease					0.243					0.145
Left main artery disease	10 (3.8)	12 (4.5)	8 (3.0)	2 (0.8)		6 (2.3)	14 (5.4)	14 (5.4)	6 (2.3)	
2-vessel disease	82 (30.9)	80 (30.1)	73 (27.4)	76 (28.7)		60 (23.0)	47 (18.0)	38 (14.6)	47 (18.1)	
3-vessel disease	38 (14.3)	29 (10.9)	37 (13.9)	33 (12.5)		59 (22.6)	62 (23.8)	48 (18.4)	57 (21.9)	
**Echocardiography**										
Admission LVEF (%)	55.0 (50.0-60.0)	55.0 (49.0-58.0)	53.0 (47.0-58.0)	53.0 (47.0-58.0)	**1.00E-06**	56.0 (52.0-60.0)	55.0 (50.0-60.0)	55.0 (50.0-60.0)	55.0 (47.0-58.0)	**2.20E-05**
**Infarct size**										
CK-MB-based estimation(ng*h/ml)	4445.1 (1564.4-6407.6)	4863.4 (1560.4-6921.1)	5277.7 (2752.1-7444.9)	5577.4 (2619.8-7845.5)	**7.31E-07**	4253.4 (2073.6-6108.0)	4566.5 (2432.1-60401.0)	4357.6 (2380.1-6178.0)	4534.6 (2522.7-7422.7)	**0.006**
**Medication at discharge**										
Aspirin	264 (99.6)	257 (96.6)	261 (98.1)	256 (96.6)	0.629	257 (98.5)	254 (97.3)	248 (95.0)	251 (96.5)	0.095
P2Y12 receptor Inhibitor	265 (100.0)	261 (98.1)	262 (98.5)	257 (97.0)	\	253 (96.9)	244 (93.5)	230 (88.1)	237 (91.2)	**0.002**
Statin	261 (98.5)	250 (94.0)	256 (96.2)	252 (95.1)	0.899	253 (96.9)	248 (95.0)	245 (93.9)	244 (93.8)	0.087
ACEI or ARB	156 (58.9)	145 (54.5)	166 (62.4)	139 (52.5)	0.641	114 (43.7)	122 (46.7)	104 (39.8)	109 (41.9)	0.375
Beta-blockers	201 (75.8)	192 (72.2)	191 (71.8)	199 (75.1)	0.744	176 (67.4)	183 (70.1)	172 (65.9)	198 (76.2)	0.085
MRA	41 (15.4)	50 (18.9)	48 (18.0)	36 (13.6)	0.635	19 (5.7)	18 (9.7)	19 (6.3)	14 (6.4)	0.151

ACEI, angiotensin-converting enzyme inhibitor; ARB, angiotensin-receptor blocker; BNP, B-type natriuretic peptide; CAD, coronary artery disease; cTnI, cardiac troponin I; DBP, diastolic blood pressure; HDL, high-density lipoprotein; hs-CRP, high-sensitivity C-reactive protein; LDL, low-density lipoprotein; LVEF, left ventricular ejection fraction; MRA, mineralocorticoid receptor antagonist; P2Y12 receptor Inhibitor included clopidogrel and ticagrelor; SBP, systolic blood pressure.

Data were presented as absolute numbers (percentages) or medians (interquartile ranges). Test for trend based on quartiles of S100A8/A9 levels. All tests were two-sided and $P < 0.05$ was considered as significant. Source data are provided in the Source Data File. Bold indicated $P<0.05$.

Supplementary Table 14. 50 S100A9 *cis*- protein quantitative trait loci (pQTLs) and their association with S100A9 in the genome-wide association studies (GWAS) summary statistic

Chrom	Pos (GRCh38)	rsids	effectAllele	otherAllele	Beta	SE	*P*-value	N	ImpMAF	MAF in Asian
chr1	153344712	NA	C	G	0.2869	0.1442	0.0466	35363	0.00111	0.00000
chr1	153345524	rs552917187	G	A	-0.1508	0.0463	0.0011	35363	0.00791	0.00000
chr1	153347960	rs779301061	G	GGA	-0.4162	0.2022	0.0396	35363	0.00036	0.00008
chr1	153351365	rs115697787	A	G	-0.0932	0.0448	0.0374	35362	0.00836	0.00000
chr1	153353113	rs745415623	T	C	1.1565	0.4320	0.0074	35363	0.00013	0.00000
chr1	153353210	rs780661487	G	C	0.1770	0.0903	0.0499	35363	0.00202	0.00000
**chr1**	**153356544**	**rs59961408**	**A**	**G**	**-0.0255**	**0.0123**	**0.0381**	**35361**	**0.12511**	**0.20400**
chr1	153356930	rs769941685	A	G	-0.2531	0.1283	0.0485	35360	0.00120	0.00000
**chr1**	**153357702**	**rs12119788**	**A**	**G**	**0.0371**	**0.0132**	**0.0048**	**35360**	**0.10879**	**0.08500**
chr1	153358301	rs112531265	A	G	-0.0878	0.0386	0.0228	35360	0.01117	0.00000
chr1	153358798	rs747411606	A	G	-0.3138	0.1345	0.0196	35360	0.00081	0.00000
**chr1**	**153359373**	**rs2916193**	**A**	**G**	**-0.0477**	**0.0094**	**0.0000**	**35360**	**0.25783**	**0.34600**
chr1	153359680	NA	CTT	CTT	0.0327	0.0090	0.0003	35350	0.35677	0.00000
chr1	153359680	rs1234133033	C	CTT	-0.1671	0.0845	0.0481	35350	0.00227	0.00000
chr1	153359680	rs1168990096, rs34039196	CT	CTT	-0.0471	0.0097	0.0000	35350	0.25293	0.00000
chr1	153361148	rs537228847	T	C	-0.2199	0.1002	0.0282	35360	0.00156	0.00000
**chr1**	**153361324**	**rs2070864**	**G**	**A**	**-0.0456**	**0.0092**	**0.0000**	**35360**	**0.27329**	**0.02000**
chr1	153361732	rs952053618	A	G	-0.4306	0.2077	0.0382	35360	0.00033	0.00000
**chr1**	**153362049**	**rs1560833**	**A**	**G**	**-0.0466**	**0.0093**	**0.0000**	**35360**	**0.26674**	**0.42500**
**chr1**	**153362908**	**rs1560832**	**A**	**G**	**-0.0466**	**0.0093**	**0.0000**	**35360**	**0.26668**	**0.39200**
**chr1**	**153363542**	**rs724781**	**G**	**C**	**-0.0468**	**0.0093**	**0.0000**	**35360**	**0.26681**	**0.34800**
chr1	153364102	NA	CAAAAAAAAAAA	CAAAAAAAAAAA	0.0212	0.0107	0.0478	35355	0.18718	0.00000

chr1	153364102	rs1478401322, rs767275639	CAAAAAAAA	CAAAAAAAAAAA	-0.0527	0.0106	0.0000	35355	0.18232	0.00000
chr1	153364102	rs397863495, rs398049600, rs5777859	CAAAAAAAAAA	CAAAAAAAAAAA	0.0185	0.0084	0.0273	35355	0.46769	0.00000
chr1	153364645	rs74807144	G	A	-0.1155	0.0266	0.0000	35360	0.02353	0.00000
**chr1**	**153365467**	**rs3014874**	**A**	**G**	**-0.0537**	**0.0094**	**0.0000**	**35360**	**0.25791**	**0.30200**
chr1	153365872	rs191112671	A	G	-0.1639	0.0696	0.0185	35360	0.00372	0.00000
**chr1**	**153366275**	**rs3014875**	**A**	**G**	**-0.0282**	**0.0086**	**0.0010**	**35360**	**0.34949**	**0.11700**
chr1	153366824	rs540566308	C	CAAG	-0.1351	0.0552	0.0144	35361	0.00569	0.00000
**chr1**	**153370099**	**rs58644524**	**T**	**C**	**0.0273**	**0.0133**	**0.0397**	**35361**	**0.10771**	**0.09400**
**chr1**	**153370702**	**rs3014878**	**T**	**C**	**0.0275**	**0.0133**	**0.0384**	**35361**	**0.10772**	**0.07900**
**chr1**	**153371241**	**rs35195593**	**G**	**GC**	**0.0276**	**0.0133**	**0.0375**	**35361**	**0.10784**	**0.01000**
**chr1**	**153371405**	**rs3014879**	**G**	**A**	**0.0262**	**0.0132**	**0.0473**	**35361**	**0.10922**	**0.09800**
**chr1**	**153371466**	**rs12033317**	**A**	**G**	**0.0276**	**0.0133**	**0.0375**	**35361**	**0.10784**	**0.09600**
chr1	153371894	NA	CATGC	CATGC	-0.0277	0.0133	0.0372	35361	0.10763	0.00000
chr1	153371894	rs3014880	CATGG	CATGC	0.0277	0.0133	0.0372	35361	0.10763	
**chr1**	**153372160**	**rs3006475**	**C**	**A**	**0.0274**	**0.0133**	**0.0388**	**35362**	**0.10780**	**0.08050**
**chr1**	**153372439**	**rs3014881**	**G**	**A**	**0.0260**	**0.0132**	**0.0488**	**35362**	**0.10916**	**0.10500**
chr1	153373217	NA	ATATATATA	ATATATATA	0.1288	0.0424	0.0024	35362	0.00921	0.00000
chr1	153373217	rs542546103	ATATATA	ATATATATA	-0.1544	0.0476	0.0012	35362	0.00747	0.00000
**chr1**	**153373435**	**rs2916191**	**C**	**T**	**0.0260**	**0.0132**	**0.0486**	**35362**	**0.10917**	**0.08200**
**chr1**	**153373787**	**rs4772**	**G**	**A**	**0.0260**	**0.0132**	**0.0485**	**35362**	**0.10917**	**0.07100**
**chr1**	**153376634**	**rs3006476**	**A**	**C**	**0.0272**	**0.0133**	**0.0409**	**35362**	**0.10751**	**0.02900**
chr1	153377394	rs577781525	ATCCTGAGATGTT	ATCCTGAGATGT	0.1063	0.0460	0.0208	35360	0.00877	0.00000
chr1	153377731	rs141832834	A	AG	0.0271	0.0133	0.0419	35362	0.10740	0.01000
**chr1**	**153379651**	**rs3014885**	**T**	**C**	**0.0272**	**0.0133**	**0.0409**	**35362**	**0.10754**	**0.10500**
chr1	153379784	NA	ACACCTAGGGTGGCGGCGGCTCCTTGGCAG	ACACCTAGGGTGGCGGCGGCTCCTTGGCAG	-0.0259	0.0132	0.0498	35362	0.10892	0.00000

chr1	153379784	rs374815343, rs555202163	A	ACACCTAG GGTGGCGG CGGCTCCT TGGCAG	0.0259	0.0132	0.0498	35362	0.10892	0.00000
chr1	153380435	NA	CGTCTCGG	CGTCTCGG	-0.0279	0.0132	0.0346	35362	0.10976	0.00000
chr1	153380435	rs377316404	CGTCTCGGA	CGTCTCGG	0.0279	0.0132	0.0347	35362	0.10975	0.00000

The 20 SNPs with MAF > 0.01 in the East Asian population are in bold. All tests were two-sided and $P < 0.05$ was considered as significant.

Supplementary Table 15. Seventeen pQTLs show associations with mRNA expression of S100A8 in the GTEx portal

SNP Id	Variant Id	*P*-value	NES	Tissue
rs2916193	chr1_153359373_G_A_b38	0.00000032	-0.062	Whole Blood
rs2070864	chr1_153361324_A_G_b38	0.000015	-0.049	Whole Blood
rs1560833	chr1_153362049_G_A_b38	0.000000048	-0.065	Whole Blood
rs1560832	chr1_153362908_G_A_b38	0.00000043	-0.057	Whole Blood
rs724781	chr1_153363542_C_G_b38	0.000000029	-0.066	Whole Blood
**rs3014874**	**chr1_153365467_G_A_b38**	**3.6E-09**	**-0.07**	**Whole Blood**
rs58644524	chr1_153370099_C_T_b38	0.000025	0.073	Whole Blood
rs3014878	chr1_153370702_C_T_b38	0.000018	0.076	Whole Blood
rs35195593	chr1_153371241_GC_G_b38	0.000031	0.073	Whole Blood
rs3014879	chr1_153371405_A_G_b38	0.000031	0.073	Whole Blood
rs12033317	chr1_153371466_G_A_b38	0.000064	0.071	Whole Blood
rs3006475	chr1_153372160_A_C_b38	0.0000097	0.075	Whole Blood
rs3014881	chr1_153372439_A_G_b38	0.000031	0.073	Whole Blood
rs2916191	chr1_153373435_T_C_b38	0.000013	0.076	Whole Blood
rs4772	chr1_153373787_A_G_b38	0.000013	0.076	Whole Blood
rs3006476	chr1_153376634_C_A_b38	0.0000084	0.076	Whole Blood
rs3014885	chr1_153379651_C_T_b38	0.000011	0.076	Whole Blood

NES, normalized effect size; SNP, single nucleotide polymorphism. NES is computed as the effect of the alternative allele relative to the reference allele.

All tests were two-sided and $P < 0.05$ was considered as significant. The SNP with the most significant P-value is in bold.

Supplementary Table 16. Seventeen pQTLs show associations with mRNA expression of S100A9 in the GTEx portal

SNP Id	Variant Id	*P*-value	NES	Tissue
rs2916193	chr1_153359373_G_A_b38	5.00E-11	-0.076	Whole Blood
rs2070864	chr1_153361324_A_G_b38	0.0000038	-0.049	Whole Blood
rs1560833	chr1_153362049_G_A_b38	2.40E-10	-0.071	Whole Blood
rs1560832	chr1_153362908_G_A_b38	2.80E-09	-0.063	Whole Blood
rs724781	chr1_153363542_C_G_b38	4.60E-11	-0.074	Whole Blood
**rs3014874**	**chr1_153365467_G_A_b38**	**2.80E-14**	**-0.085**	**Whole Blood**
rs58644524	chr1_153370099_C_T_b38	2.60E-09	0.097	Whole Blood
rs3014878	chr1_153370702_C_T_b38	6.30E-10	0.1	Whole Blood
rs35195593	chr1_153371241_GC_G_b38	5.80E-10	0.1	Whole Blood
rs3014879	chr1_153371405_A_G_b38	5.80E-10	0.1	Whole Blood
rs12033317	chr1_153371466_G_A_b38	1.50E-09	0.1	Whole Blood
rs3006475	chr1_153372160_A_C_b38	1.30E-10	0.1	Whole Blood
rs3014881	chr1_153372439_A_G_b38	5.80E-10	0.1	Whole Blood
rs2916191	chr1_153373435_T_C_b38	4.20E-11	0.11	Whole Blood
rs4772	chr1_153373787_A_G_b38	4.20E-11	0.11	Whole Blood
rs3006476	chr1_153376634_C_A_b38	2.80E-10	0.1	Whole Blood
rs3014885	chr1_153379651_C_T_b38	1.50E-09	0.098	Whole Blood

NES, normalized effect size; SNP, single nucleotide polymorphism. NES is computed as the effect of the alternative allele relative to the reference allele.

All tests were two-sided and $P < 0.05$ was considered as significant. The SNP with the most significant P-value is in bold.

Supplementary Table 17. Clinical characteristics in patients with AMI from UK Biobank (UKB)

Variable	UKB (n = 1144)		*P-value*
	HF events (n = 224)	No-HF events (n = 920)	
Age (years)	56.0 (52.6–59.2)	55.7 (51.6–58.4)	0.062
Male sex (%)	157 (70.1)	649 (70.5)	0.894

Data were presented as absolute numbers (percentages) or medians (interquartile ranges). Categorical variables were analyzed using a two-sided χ^2 or Fisher's exact test. Continuous non-parametric data were analyzed using a two-sided the Mann–Whitney U test. $P < 0.05$ was considered as significant. Source data are provided in the Source Data File.

Supplementary Fig. 1 Enrollment flowchart in the discovery and validation cohorts

a discovery cohort (n = 1062). **b** validation cohort (n =1043). AMI, acute myocardial infarction; HF, heart failure; PCI, percutaneous coronary intervention.

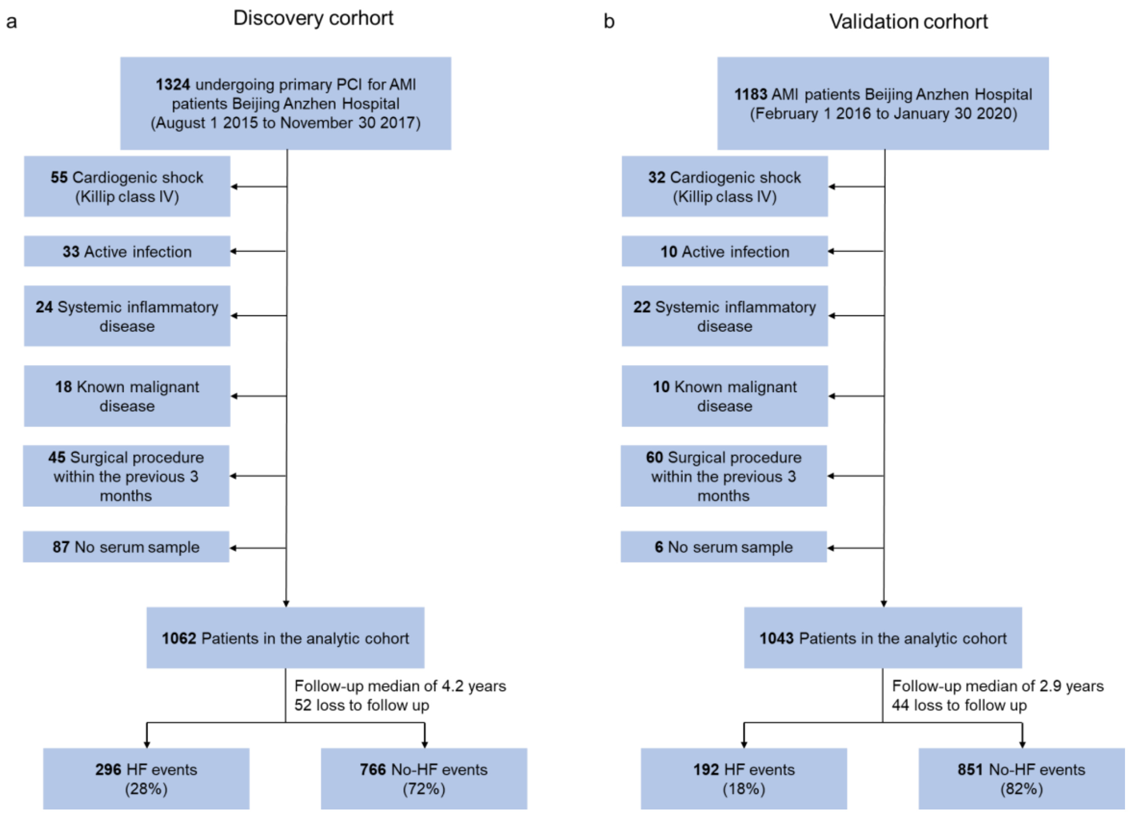

Supplementary Fig. 2 Proteomic study flowchart

AMI, acute myocardial infarction; DEPs, differentially expressed proteins; HC, healthy control, HF, heart failure.

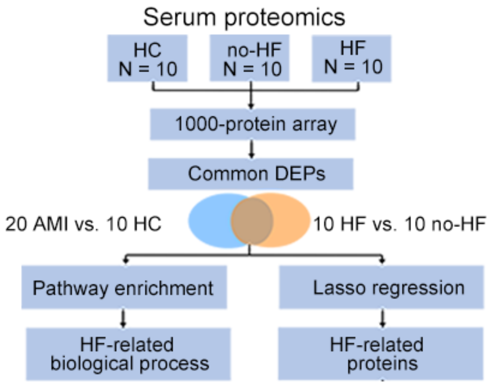

Supplementary Fig. 3 HF-associated changes in serum proteomics

a Heat map of 134 common differentially expressed proteins (DEPs) in healthy controls (HCs), patients with AMI without HF (no-HF), and patients with AMI with HF (HF). **b** The top ten enriched pathways detected using the Reactome Pathway Database indicating the biological significance of 134 common DEPs. $P < 0.05$ was considered as significant.

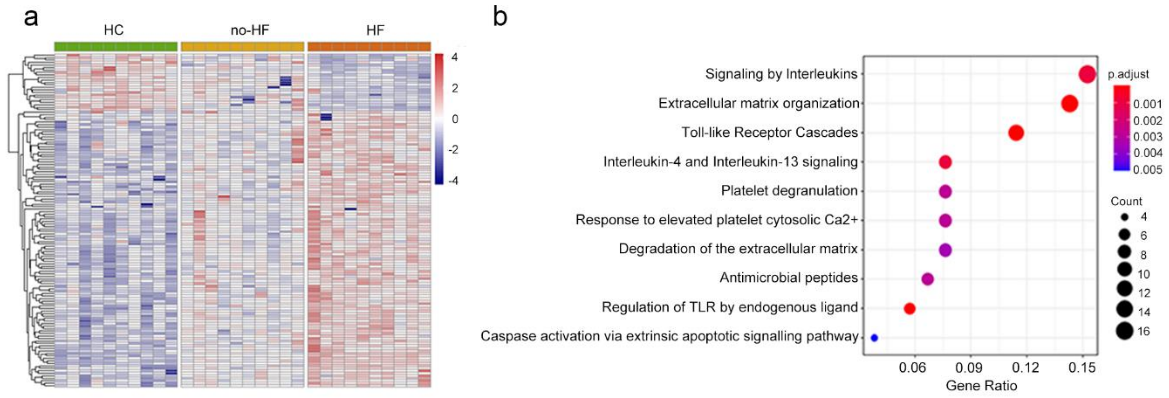

Supplementary Fig. 4 least absolute shrinkage and selection operator (LASSO) analysis to select the optimum candidate proteins

a Plots of the LASSO regression coefficients for different penalty parameter values. **b** Cross-validation plot of penalty terms. **c** Estimated coefficients for the LASSO regression of candidate proteins.

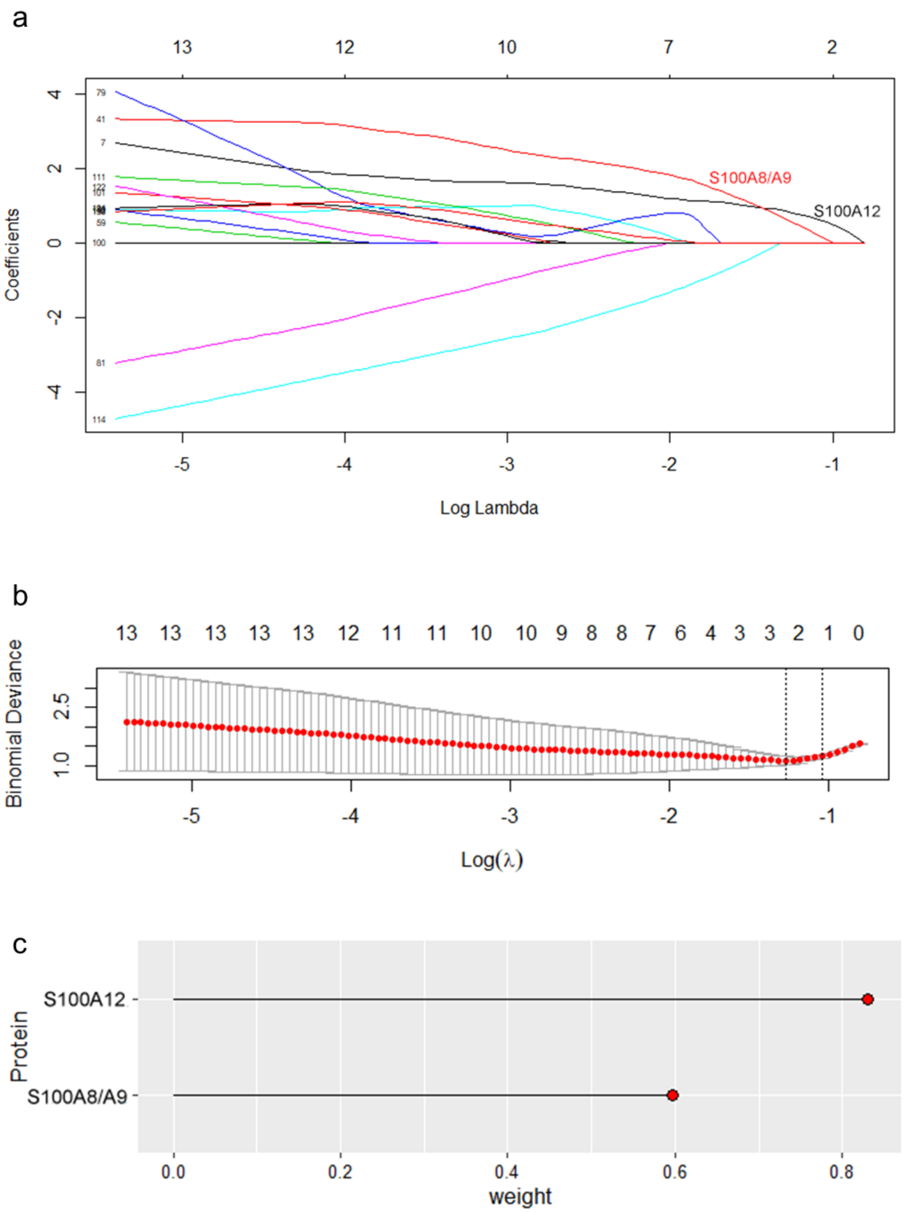

Supplementary Fig. 5 Predictive values of S100A12 in the discovery cohort

a S100A12 levels were measured upon admission to the discovery cohort. Red: Patients who experienced HF events; blue: Patients who did not experience HF events. The scatter plots in this figure show the median (center line), 25th, and 75th percentile (lower and upper boundary). Differences between the two groups were analyzed using a two-sided Wilcoxon-test. $P < 0.05$ was considered as significant. **b** Unadjusted and adjusted HRs from Cox proportional hazards regression analysis for S100A12 levels at admission in the discovery cohort (n=1062), which are indicated with the points and the bars showing the 95% confidence interval. Model 1: adjusted for age and sex; Model 2: adjusted for model 1+systolic blood pressure, Killip classification at admission, fasting glucose, creatinine, and left main artery disease; Model 3: adjusted for model 2+neutrophil count, cardiac troponin I, B-type natriuretic peptide, C-reactive protein, left ventricular ejection fraction at admission, and estimated infarct size (CK-MB $AUC_{0\text{-}72}$). P values reported are two-tailed from COX proportional hazard regression analyses. $P < 0.05$ was considered as significant. HF, heart failure; HRs, hazard ratios.

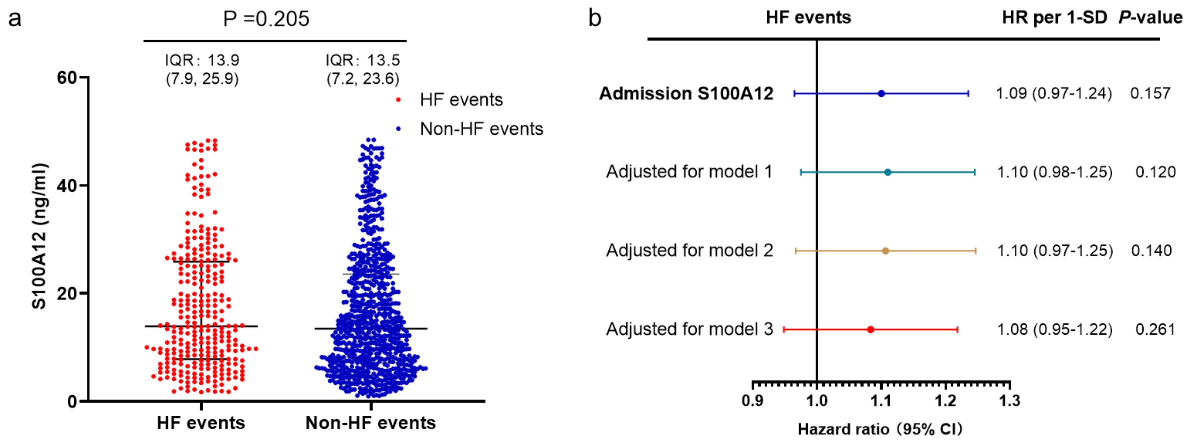

Supplementary Fig. 6 Multiple-adjusted hazard ratios of HF events associated with HF-related proteins

Possible nonlinear relationships between HF-related proteins and HF events were examined with restricted cubic splines in discovery cohort (n=1062). Hazard ratios of S100A8/A9 (**a**) and S100A12 (**b**) were adjusted for age, sex, systolic blood pressure, Killip classification at admission, fasting glucose, creatinine, left main artery disease, neutrophil count, cardiac troponin I, B-type natriuretic peptide, C-reactive protein, left ventricular ejection fraction at admission, and estimated infarct size (CK-MB AUC0-72).

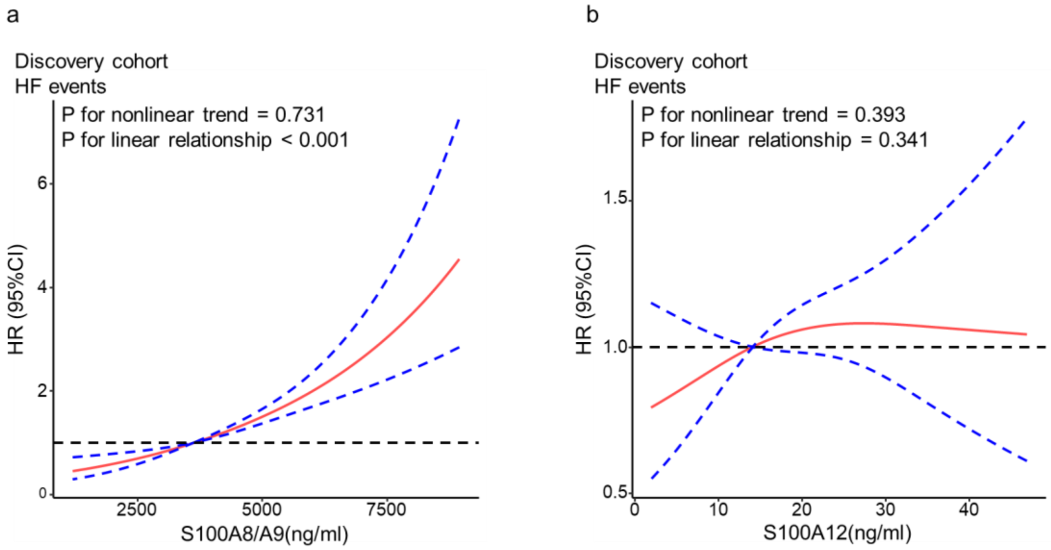

Supplementary Fig. 7 Subgroup analysis of S100A8/A9 and S100A12 prognostic values

The adjusted variables for S100A8/A9 (**a**) and S100A12 (**b**) included age, sex, systolic blood pressure, Killip classification at admission, fasting; creatinine, left main artery disease, neutrophil count, cardiac troponin I, B-type natriuretic peptide, C-reactive protein levels, left ventricular ejection fraction at admission, and estimated infarct size (CK-MB AUC_{0-72}). Boxes represent HR. Error bars represent 95% CI. P values reported are two-tailed from COX proportional hazard regression analyses. HR, hazard ratio.

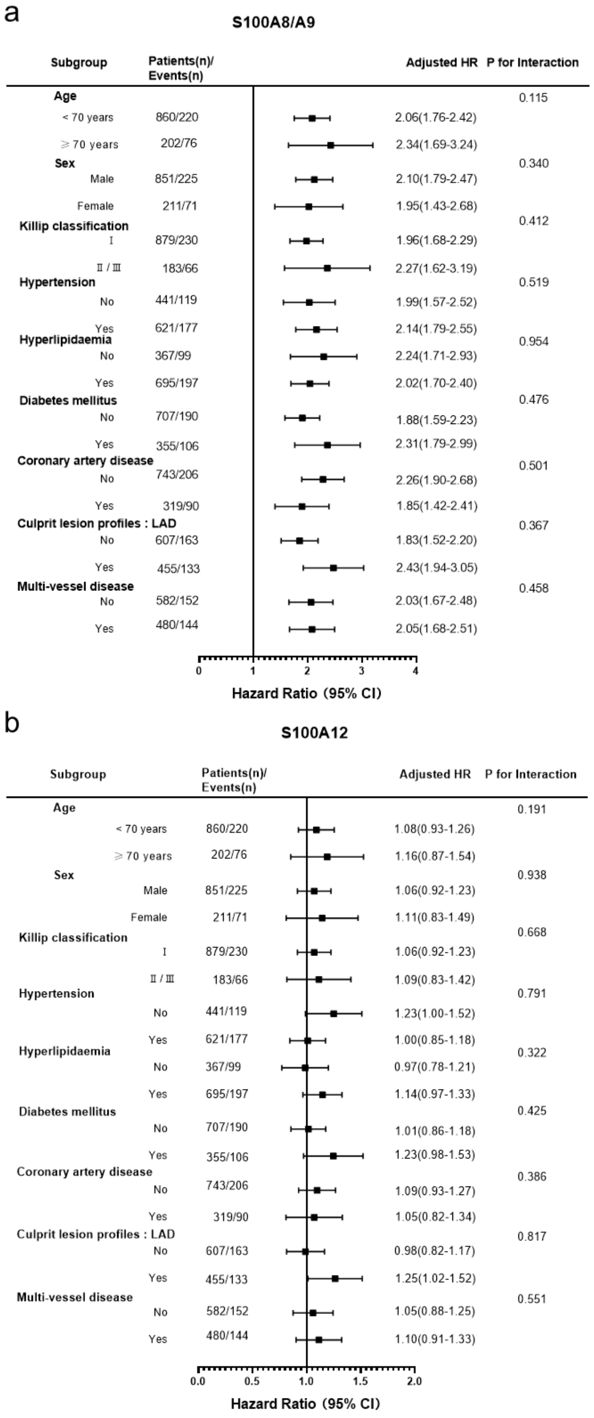

Supplementary Fig. 8 Decision curve analysis of combined S100A8/A9 and reference model *vs.* reference model alone for the HF events

The reference models included age, sex, systolic blood pressure (SBP), Killip classification at admission, fasting glucose, creatinine, left main artery disease, neutrophil count, cardiac troponin I, B-type natriuretic peptide, C-reactive protein levels, left ventricular ejection fraction at admission, and estimated infarct size (CK-MB $AUC_{0\text{-}72}$) in the discovery (**a**) and validation cohorts (**b**).

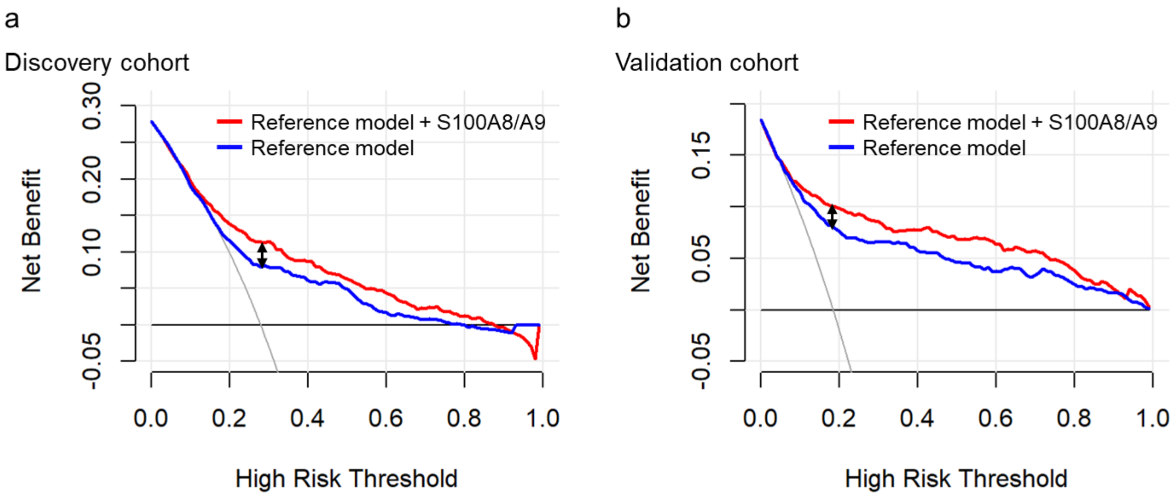

Supplementary Fig. 9 S100A8/A9 plasma concentrations in each Killip class on admission in combined cohorts

S100A8/A9 levels were show in combined cohorts according to Killip class I, II and III. Green: Patients with Killip class I; blue: Patients with Killip class II; Red: Patients with Killip class III. The scatter plots show the median (center line), 25th, and 75th percentile (lower and upper boundary). Differences between the two groups were analyzed using a two-sided Wilcoxon-test. P < 0.05 was considered as significant.

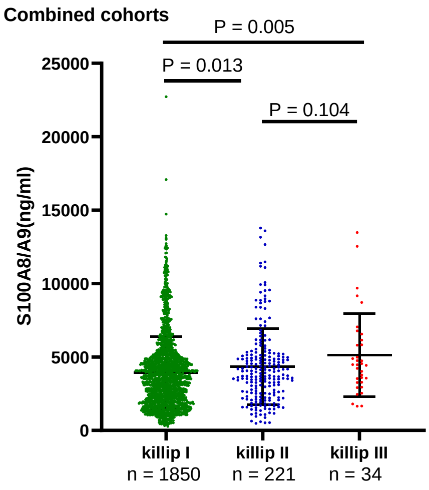

Supplementary Fig. 10 Correlation between serum S100A8/A9 levels and S100A9 levels

a Scatter plot of the correlation between S100A8/A9 levels and S100A9. P values reported are two-tailed from pearson correlation. P < 0.05 was considered as significant. **b-c** Distribution of S100A9 (**b**) and S100A8/A9 (**c**) in HC (n = 24), patients with AMI without HF (n=24), and patients with AMI with HF (n = 24). Differences between the two groups were analyzed using a two-sided Wilcoxon-test. P < 0.05 was considered as significant.

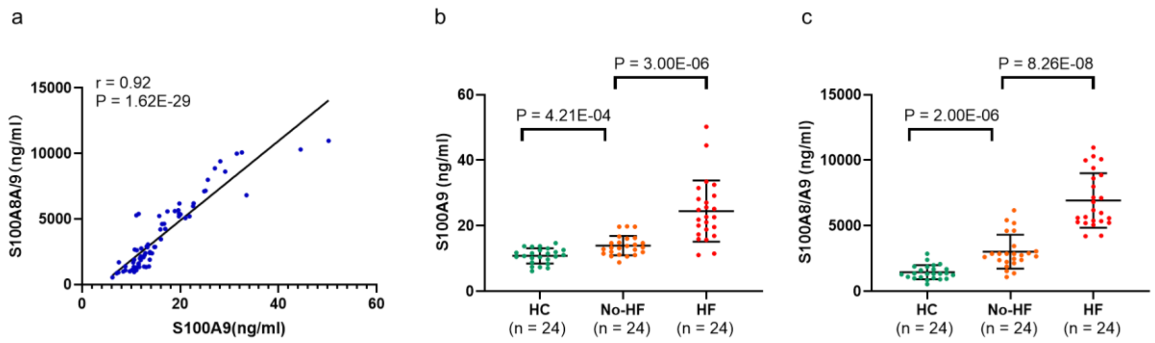

Supplementary Fig. 11 S100A8/A9 genetic score construction

a Mendelian randomization (MR) study design. **b** Twenty cis-pQTLs selecting within 20 kb of either side of S100A9 and their linkage disequilibrium relationship calculated by Haploview software using CHB data as reference. The number in each cell is R^2 of its corresponding two SNPs, which is a parameter indicating the degree of linkage disequilibrium between two SNPs. The larger R^2, the stronger the degree of linkage disequilibrium. The red cells without number mean the $R^2 = 1$.

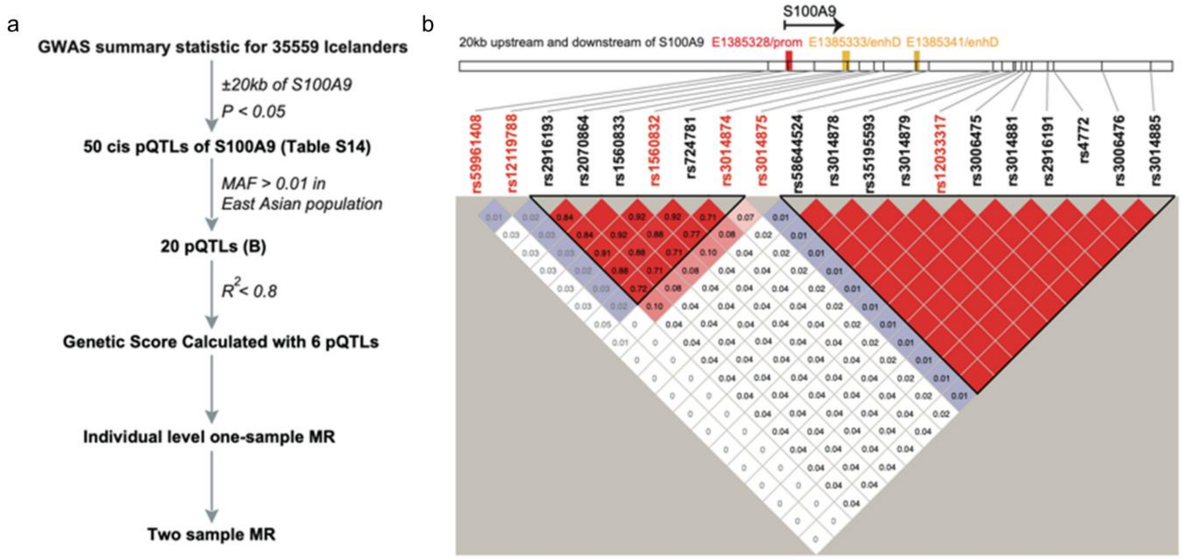

Supplementary Fig. 12 Association between S100A8/A9 genetic score and S100A8/A9 plasma concentrations in the validation cohort.

a Distribution of S100A8/A9 plasma concentrations in different S100A8/A9 genetic score groups. Differences between the two groups were analyzed using a two-sided Wilcoxon-test. $P < 0.05$ was considered as significant. **b** Scatter plot correlation of S100A8/A9 plasma concentrations and S100A8/A9 genetic score. P values reported are two-tailed from pearson correlation. $P < 0.05$ was considered as significant.

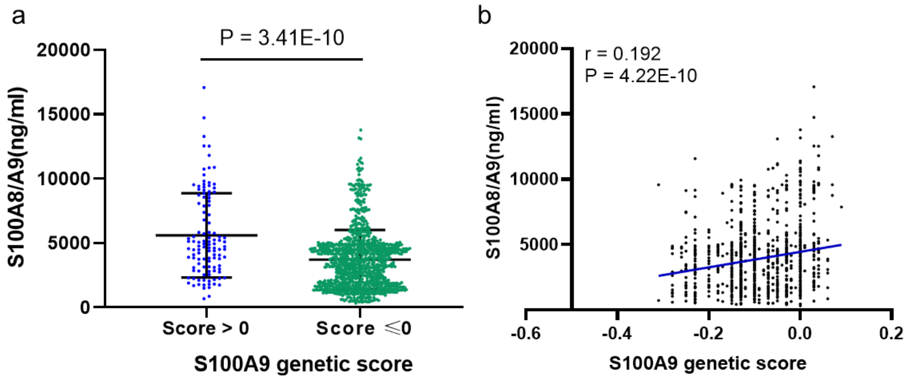

Supplementary Fig. 13 Diagram of two-sample MR framework in the present study

AMI, acute myocardial infarction; HF, heart failure; IVW, inverse variance-weighted; MR, Mendelian randomization; pQTL, protein quantitative trait loci; SNPs, single nucleotide polymorphisms; UKB, UK Biobank.

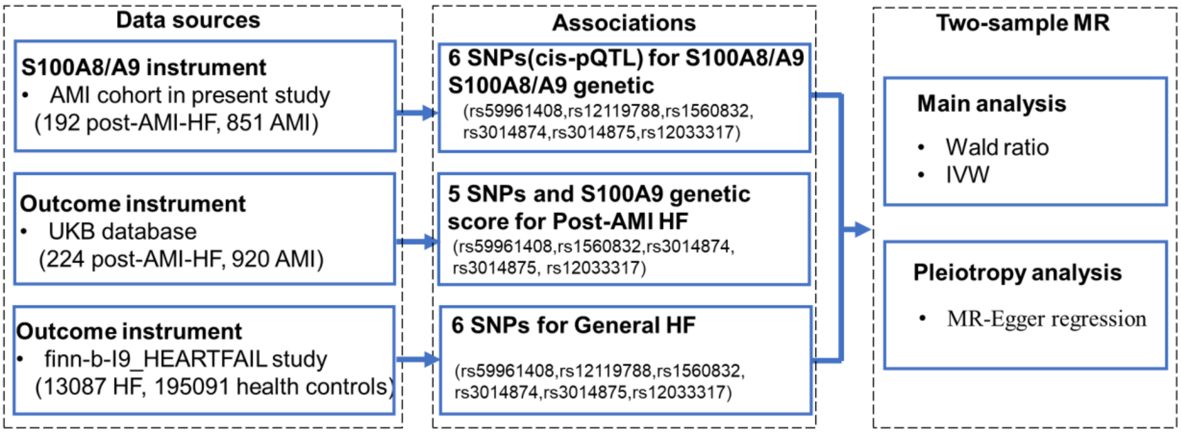

Supplementary Fig. 14 Dose-response curves showing the causal effect of S100A8/A9 levels on post- myocardial infarction /general HF

The strength of the association between HF events and each S100A9 SNPs on the y-axis against the association between S100A8/A9 levels and each SNP on the x-axis in validation cohort (192 HF events, 851 No-HF events) (**a**), UK Biobank (224 HF events, 920 No-HF events) (**b**) and, finn-b-I9_HEARTFAIL study (13,087 patients with HF, 19,5091 controls) (**c**). The slopes of line represent the causal effect of inverse-variance weighted.

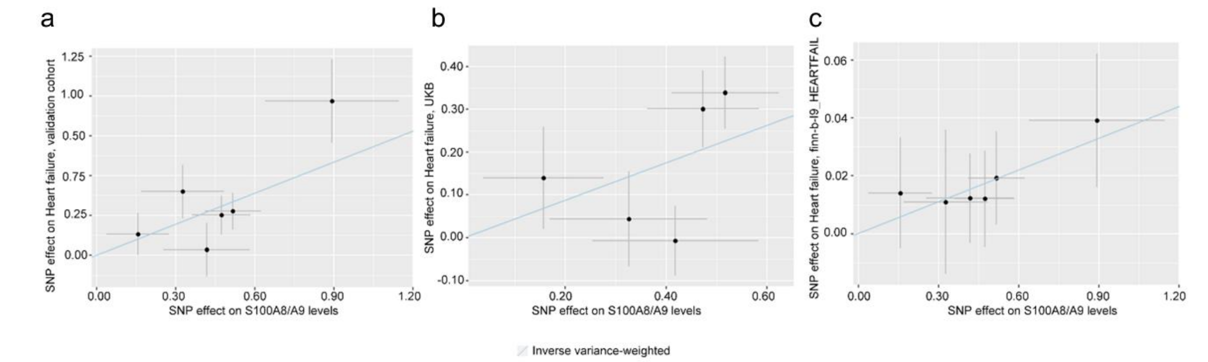

Supplementary Fig. 15 LocusZoom plots of the pQTLs and GWAS signals within 20 kb of either side of S100A9.

a LocusZoom plot indicating pQTL of S100A9 in serum. **b** LocusZoom plot indication GWAS signals for post-AMI HF in UKB cohort. **c** LocusZoom plot indication GWAS signals for general HF in finn-b-19 HEARTFAIL.

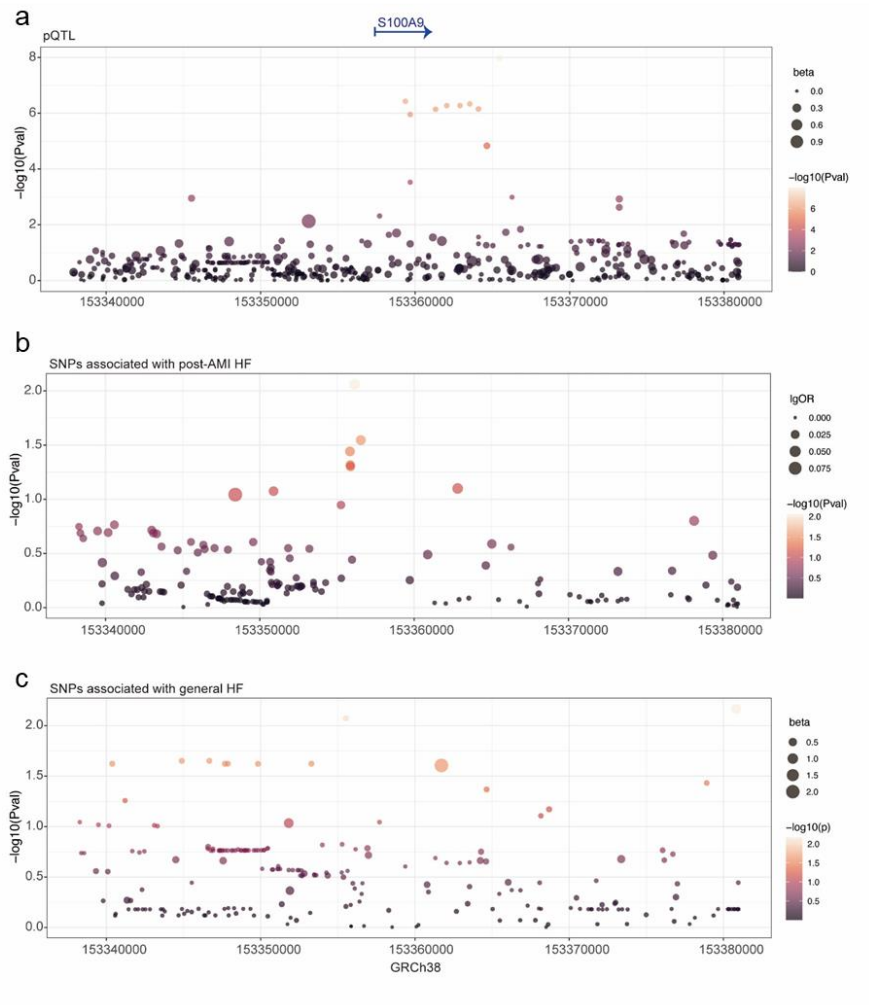

Supplementary Fig. 16 Estimation of infarct size in discovery and validation cohorts. Measurement of infarct size by 72-h creatine kinase-MB isoenzyme (**a, b**) release. CK-MB, Creatine kinase-MB isoenzyme.

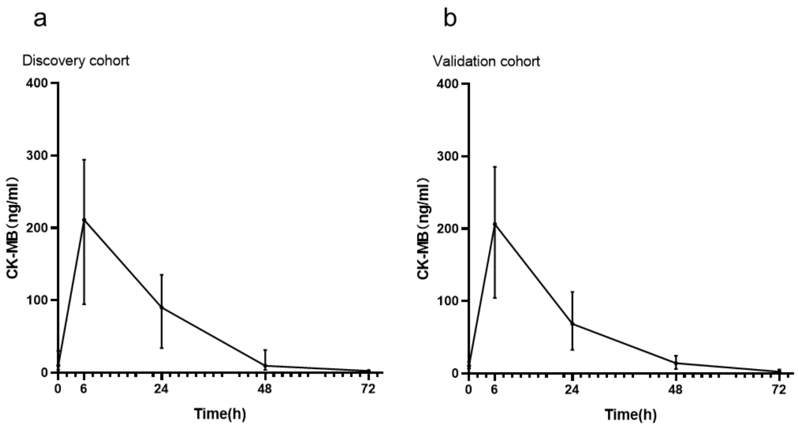

Supplement: Supplementary file 1 — Supplementary Information [file 41467_2024_46973_MOESM1_ESM.pdf]
