# Supplementary material for: S100A8/A9 as a prognostic biomarker with causal effects for post-acute myocardial infarction heart failure
Source: Nat Commun. 2024 Mar 27;15:2701. doi: 10.1038/s41467-024-46973-7 (PMC10973499; doi:10.1038/s41467-024-46973-7)
Supplement: Supplementary file 3 — Description of Additional Supplementary Files [file 41467_2024_46973_MOESM3_ESM.pdf]

Description of Additional Supplementary File

File Name: Supplementary Data 1

Description: The human antibody array containing 1000 proteins from HCs and patients with AMI with or without HF events at admission (n = 10 per group) in the step 1.
