# Supplementary material for: S100A8/A9 as a prognostic biomarker with causal effects for post-acute myocardial infarction heart failure
Source: Nat Commun. 2024 Mar 27;15:2701. doi: 10.1038/s41467-024-46973-7 (PMC10973499; doi:10.1038/s41467-024-46973-7)
Supplement: Supplementary file 5 — Reporting Summary [file 41467_2024_46973_MOESM5_ESM.pdf]

nature portfolio

Corresponding author(s): Yulin Li

Last updated by author(s): Mar 8, 2024

Reporting Summary

Nature Portfolio wishes to improve the reproducibility of the work that we publish. This form provides structure for consistency and transparency in reporting. For further information on Nature Portfolio policies, see our Editorial Policies and the Editorial Policy Checklist.

Statistics

For all statistical analyses, confirm that the following items are present in the figure legend, table legend, main text, or Methods section.

n/a	Confirmed	
☐	☒	The exact sample size (*n*) for each experimental group/condition, given as a discrete number and unit of measurement
☐	☒	A statement on whether measurements were taken from distinct samples or whether the same sample was measured repeatedly
☐	☒	The statistical test(s) used AND whether they are one- or two-sided<br>*Only common tests should be described solely by name; describe more complex techniques in the Methods section.*
☐	☒	A description of all covariates tested
☐	☒	A description of any assumptions or corrections, such as tests of normality and adjustment for multiple comparisons
☐	☒	A full description of the statistical parameters including central tendency (e.g. means) or other basic estimates (e.g. regression coefficient) AND variation (e.g. standard deviation) or associated estimates of uncertainty (e.g. confidence intervals)
☐	☒	For null hypothesis testing, the test statistic (e.g. *F*, *t*, *r*) with confidence intervals, effect sizes, degrees of freedom and *P* value noted<br>*Give P values as exact values whenever suitable.*
☒	☐	For Bayesian analysis, information on the choice of priors and Markov chain Monte Carlo settings
☒	☐	For hierarchical and complex designs, identification of the appropriate level for tests and full reporting of outcomes
☐	☒	Estimates of effect sizes (e.g. Cohen's *d*, Pearson's *r*), indicating how they were calculated

Our web collection on statistics for biologists contains articles on many of the points above.

Software and code

Policy information about availability of computer code

Data collection	No software was used for data collection.
Data analysis	Analyses were performed using IBM SPSS software (version 24.0; SPSS Inc., Chicago, IL, USA) or R (version 4.1.1; R Foundation for Statistical Computing, Vienna, Austria).We used the glmnet package, clusterProfiler package, ReactomePA package, cmprsk package, ivreg package, TwoSampleMR package, coloc package in R programming.

For manuscripts utilizing custom algorithms or software that are central to the research but not yet described in published literature, software must be made available to editors and reviewers. We strongly encourage code deposition in a community repository (e.g. GitHub). See the Nature Portfolio guidelines for submitting code & software for further information.

Data

Policy information about availability of data

All manuscripts must include a data availability statement. This statement should provide the following information, where applicable:

- Accession codes, unique identifiers, or web links for publicly available datasets
- A description of any restrictions on data availability
- For clinical datasets or third party data, please ensure that the statement adheres to our policy

The raw data supporting the findings of the study have been provided in source data. Due to ethical and legal restrictions, the clinical data are not publicly available. Any individual affiliated with an academic institution may request access to the clinical data from Yulin Li, PhD (lyllyl_1111@163.com) for research purposes. This

includes submitting a proposal to the management team, where upon approval, data will be provided with a signed data access agreement (refer to the supplementary material file titled 'Data Access Agreement'). The timeframe for responding to an access to information is a 20-working day from the gate of receipt. Source data are provided with this paper.
Three public data were used in the Mendelian randomization analysis. The GWAS summary statistics for S100A9 “5339_49_S100A9_calgranulin_B.txt.gz” be downloaded at https://www.decode.com/summarydata/.The data of AMI patients in UK Biobank cohort were download from at https://www.ukbiobank.ac.uk/ and received under the data request application no.68808. The heart failure GWAS of summary statistics from finn-b-I9_HEARTFAIL study are publicly available at https://gwas.mrcieu.ac.uk/datasets/finn-b-I9_HEARTFAIL/.

Research involving human participants, their data, or biological material

Policy information about studies with human participants or human data. See also policy information about sex, gender (identity/presentation), and sexual orientation and race, ethnicity and racism.

Reporting on sex and gender

No sex specific-analyses were perfomed. Both sexes were included in the analyses.

Reporting on race, ethnicity, or other socially relevant groupings

No race, ethnicity and other socially relevant specific-analyses were perfomed.

Population characteristics

Clinicopathological characteristics of AMI patients (n=24) and HCs (n=12) in step 1 are presented in Supplemental Table 1. In discovery cohort, HF patients were older; had a higher proportion of Killip classification III; higher serum creatinine, fasting glucose, neutrophil counts, and biomarkers (cTnI, BNP, hs-CRP); lower blood pressure (SBP and diastolic blood pressure) and LVEF; larger infarct size; and more left main lesions. Medications were similar between HF and no-HF patients. In validation cohort, AMI patients with HF had lower blood pressure, larger infarct size and higher creatinine, neutrophil counts, and biomarkers. Multi-vessel disease and medications were similar between HF and no-HF patients (Table 1).
Baseline information of the general population (n=588) in step 3 are presented in Supplemental Table 5.

Recruitment

For the discovery cohort, we recruited consecutive 1,324 patients with AMI at the Beijing Anzhen Hospital of Capital Medical University between August 1, 2015, and November 30, 2017. Patients with cardiogenic shock (Killip class IV), active infection, systemic inflammatory disease, known malignant disease, or surgery within the previous 3 months were excluded, while 1,062 patients were enrolled according to stringent criteria (Supplementary Fig. 1).
For the validation cohort, we recruited 1,183 patients with AMI (age: 18–45 years) from Beijing Anzhen Hospital of Capital Medical University between February 1, 2016, and January 30, 2020, with similar exclusion criteria, and finally included 1,043 patients with AMI (Supplementary Fig. 1).
HCs were recruited among individuals receiving regular physical examinations at Beijing Anzhen Hospital, and HC status was confirmed using electrocardiogram, transthoracic echocardiography, and laboratory examination to exclude MI, HF, or abnormal cardiac structure/function.

Ethics oversight

The written informed consent was obtained from all participants, and local ethics committee approved the study protocols. This study followed the Strengthening the Reporting of Observational Studies in Epidemiology reporting guideline and Declaration of Helsinki, and was registered in ClinicalTrials.gov (ID: NCT03752515).

Note that full information on the approval of the study protocol must also be provided in the manuscript.

Field-specific reporting

Please select the one below that is the best fit for your research. If you are not sure, read the appropriate sections before making your selection.

☒ Life sciences ☐ Behavioural & social sciences ☐ Ecological, evolutionary & environmental sciences

For a reference copy of the document with all sections, see nature.com/documents/nr-reporting-summary-flat.pdf

Life sciences study design

All studies must disclose on these points even when the disclosure is negative.

Sample size

Assuming HF event rate of 10% and covariates prediction up to 30% of the biomarker variance, the sample size of 913 patients could provide a 90% power to detect a biomarker hazard ratio (HR) of 1.5. Discovery cohort composed 1062 patients and validation cohort composed 1043 patients meet this sample size.

Data exclusions

Patients with cardiogenic shock (Killip class IV), active infection, systemic inflammatory disease, known malignant disease, or surgery within previous 3 months were excluded.
For the discovery cohort, we recruited consecutive 1,324 patients with AMI at the Beijing Anzhen Hospital of Capital Medical University between August 1, 2015, and November 30, 2017. Patients with cardiogenic shock (Killip class IV), active infection, systemic inflammatory disease, known malignant disease, or surgery within the previous 3 months were excluded. Further, 1,062 patients were enrolled according to stringent criteria (Supplemental Figure 1).
For the validation cohort, we recruited 1,183 patients with AMI (age: 18–45 years) from Beijing Anzhen Hospital of Capital Medical University between February 1, 2016, and January 30, 2020, with similar exclusion criteria, and finally included 1,043 patients with AMI (Supplemental Figure 1).

Replication

Biomarkers associated with post-AMI HF were identified based on proteomic data from 10 patients with AMI, 10 patients with HF post-AMI

Replication

and 10 healthy individuals. We validated the association of S100A8/A9 levels with post-AMI HF using discovery and validation cohorts detailed in the manuscript. In addition, we validated the causal association between the post-AMI HF biomarker and HF in the validation cohort, UK-Biobank and finn-b-I9_HEARTFAIL database.

Randomization

There was no randomization in the study procedure. All paticipants followed the same study procedure.

Blinding

All events were adjudicated by the consensus of 2 experienced cardiologists who were blinded to study results. Disagreements were resolved through discussion and seeking a third opinion from another blinded experienced cardiologists, as needed.

Reporting for specific materials, systems and methods

We require information from authors about some types of materials, experimental systems and methods used in many studies. Here, indicate whether each material, system or method listed is relevant to your study. If you are not sure if a list item applies to your research, read the appropriate section before selecting a response.

Materials & experimental systems

n/a	Involved in the study
☒	☐ Antibodies
☒	☐ Eukaryotic cell lines
☒	☐ Palaeontology and archaeology
☒	☐ Animals and other organisms
☐	☒ Clinical data
☒	☐ Dual use research of concern
☒	☐ Plants

Methods

n/a	Involved in the study
☒	☐ ChIP-seq
☒	☐ Flow cytometry
☒	☐ MRI-based neuroimaging

Clinical data

Policy information about clinical studies

All manuscripts should comply with the ICMJE guidelines for publication of clinical research and a completed CONSORT checklist must be included with all submissions.

Clinical trial registration

ClinicalTrials.gov Identifier: NCT03752515 "A Registry Study on Genetics and Biomarkers of Acute Coronary Syndrome (ARSGB-ACS)"

Study protocol

ClinicalTrials.gov Identifier: NCT03752515 "A Registry Study on Genetics and Biomarkers of Acute Coronary Syndrome (ARSGB-ACS)"

Data collection

The proteomic data of admission blood samples from 10 patients with AMI, 10 patients with HF post-MI, and 10 HCs who visited Anzhen Hospital and matched in gender and age was obtained by using Human Antibody Array method. The clinical information of patients was collected through the medical record.

For the discovery cohort, we recruited consecutive 1,324 patients with AMI at the Beijing Anzhen Hospital of Capital Medical University between August 1, 2015, and November 30, 2017. Patients with cardiogenic shock (Killip class IV), active infection, systemic inflammatory disease, known malignant disease, or surgery within the previous 3 months were excluded, while 1,062 patients were enrolled according to stringent criteria. The clinical information of patients was collected through the medical record system. Venous blood samples were collected on admission. Serum S100A8/A9 and S100A12 levels were detected using ELISA, and serum cTnI levels, plasma BNP levels and plasma hs-CRP levels were determined using chemiluminescence assay, Alere Triage immunoassay, urbidimetric inhibition immunoassay respectively.

For the validation cohort, we recruited 1,183 patients with AMI (age: 18–45 years) from Beijing Anzhen Hospital of Capital Medical University between February 1, 2016, and January 30, 2020, with similar exclusion criteria, and finally included 1,043 patients with AMI. The clinical information of patients was collected through the medical record system. Venous blood samples were collected on admission. The levels of S100A8/A9, cTnI, BNP and hs-CRP were detected using the same methods as the discovery cohort. In addition, SNPs were genotyped in validation cohort using TaqMan technology.

The occurrence of HF events for each patient in the two cohorts was collected by telephone interviews and review of outpatient clinics or hospitalization records every 6 to 12 months. The follow-up ended at death or termination (May 31, 2021; the maximum follow-up for both the discovery and validation cohorts).

HCs were recruited among individuals receiving regular physical examinations at Beijing Anzhen Hospital, and HC status was confirmed using electrocardiogram, transthoracic echocardiography, and laboratory examination to exclude MI, HF, or abnormal cardiac structure/function. S100A8/A9 levels and genotyping of SNPs in healthy individuals were detected using the same methods as described above.

Outcomes

The outcome was in-hospital and long-term post-discharge HF incidence. In-hospital HF included new HF onset (HF symptoms/signs after initial presentation, imaging evidence of pulmonary congestion), worsening heart failure (Killip class II progressed to III or IV, Killip class III progressed to IV), cardiogenic shock diagnosis, and in-hospital death due to heart failure or cardiogenic shock. Long-term HF included HF progression resulting in re-hospitalization and/or death due to heart failure after initial discharge.

Plants

Seed stocks	No seed stocks and other plant materials were used in this study.
Novel plant genotypes	No samples of any novel plant genotypes were used in this study.
Authentication	No seed stocks were used and novel plant genotypes were generated in this study.
